# Inference for correlated effect sizes using multiple univariate meta‐analyses

**DOI:** 10.1002/sim.6789

**Published:** 2015-11-04

**Authors:** Yong Chen, Yi Cai, Chuan Hong, Dan Jackson

**Affiliations:** ^1^ Department of Biostatistics and EpidemiologyUniversity of PennsylvaniaPhiladelphiaPennsylvania19104U.S.A.; ^2^ Division of BiostatisticsUniversity of Texas School of Public Health1200 Pressler St, HoustonTexas 77030U.S.A.; ^3^ MRC Biostatistics Unit, CambridgeCambridge Institute of Public HealthForvie Site, Robinson Way, Cambridge CB2 0SRU.K.

**Keywords:** method of moments, multivariate meta‐analysis, non‐iterative method, singular estimated covariance matrix, within‐study correlation

## Abstract

Multivariate meta‐analysis, which involves jointly analyzing multiple and correlated outcomes from separate studies, has received a great deal of attention. One reason to prefer the multivariate approach is its ability to account for the dependence between multiple estimates from the same study. However, nearly all the existing methods for analyzing multivariate meta‐analytic data require the knowledge of the within‐study correlations, which are usually unavailable in practice. We propose a simple non‐iterative method that can be used for the analysis of multivariate meta‐analysis datasets, that has no convergence problems, and does not require the use of within‐study correlations. Our approach uses standard univariate methods for the marginal effects but also provides valid joint inference for multiple parameters. The proposed method can directly handle missing outcomes under missing completely at random assumption. Simulation studies show that the proposed method provides unbiased estimates, well‐estimated standard errors, and confidence intervals with good coverage probability. Furthermore, the proposed method is found to maintain high relative efficiency compared with conventional multivariate meta‐analyses where the within‐study correlations are known. We illustrate the proposed method through two real meta‐analyses where functions of the estimated effects are of interest. © 2015 The Authors. *Statistics in Medicine* Published by John Wiley & Sons Ltd.

## 1 Introduction

Meta‐analysis, as the hallmark of evidence‐based medicine, is a statistical procedure to combine evidence from multiple studies. Conventionally, relatively simple methods for univariate meta‐analysis are used to investigate one outcome at a time. For example, the univariate DerSimonian and Laird procedure is extremely popular because of its ability to provide straightforward estimation and account for any between‐study heterogeneity 1. However, in situations where studies provide estimates that contribute to more than one of the univariate meta‐analyses in a systematic review, the quite common use of many univariate meta‐analyses is inappropriate when interest lies in making joint inferences for multiple parameters or for functions of these parameters. This is because the univariate estimates will in general be correlated and separate univariate meta‐analyses do not describe these correlations. Assuming that the modelling assumptions are appropriate, the results from multiple univariate meta‐analyses will be only valid for making inferences about each of the effects separately. To make valid joint inferences for multiple parameters, or functions of multiple parameters, we must take any correlation between the pooled estimates into account 2, Sec. 5.2.2.].

In particular, there is often interest in a function of more than one pooled estimate. For example, in meta‐analyses of diagnostic test accuracy studies, the log diagnostic odds ratio is often used as a summary of diagnostic test accuracy, which is a function of sensitivity and specificity 3. For studies with survival rates as outcomes, investigators may be interested in the difference between the overall survival rate and the disease‐free survival rate 4, 5. For comparative analysis of pharmacovigilance methods in the detection of adverse drug reactions, the *F* score is often used to offer a trade‐off between precision and recall, which is a function of sensitivity and positive predictive value 6. In many situations, multiple outcomes such as these are correlated 7, 8, 9.

One strategy for the meta‐analysis of correlated outcomes, which have received a great deal of attention recently, is multivariate meta‐analysis 2, 7, 10, 11. This type of meta‐analysis jointly analyzes multiple and possibly correlated outcomes in a single analysis. Typically, a two‐stage procedure is adopted. At the first stage, the multivariate summary measures and their covariance matrices for all studies are obtained. At the second stage, these reported summary measures are combined through an multivariate meta‐analysis model, such as the multivariate random‐effects model 2, 12. Inference can be performed using maximum likelihood (ML) or restricted maximum likelihood (REML) estimation, where the likelihood is calculated using the marginal distribution of the summary measures. Although conceptually straightforward, ML or REML estimation require iterative procedures and sometimes encounter convergence or singular estimated covariance matrix problems 13, 14. These estimation issues can lead to biased estimates of standard errors, and consequently, the confidence intervals may be too wide or too narrow 2, 11.

To avoid the computational difficulties of ML and REML estimation, several non‐iterative multivariate methods for random effects meta‐analysis have been proposed. Jackson *et al.*, 15 proposed a multivariate extension of DerSimonian and Laird's univariate method. This multivariate method only requires solving linear equations and standard matrix operations. In addition, in large samples, the inference is valid without the normality assumption. More recently, Chen *et al.*, 16 proposed an alternative matrix‐based multivariate extension of Dersimonian and Laird's method, which is also computationally simple and has the additional advantage of being invariant to linear transformations of the data. This matrix‐based multivariate method has been extended to include studies with missing outcome data and meta‐regressions 17. All of these non‐iterative methods are computationally much less demanding than ML or REML estimation and can resolve the problem of estimating a non‐positive semi‐definite between‐study covariance matrix by truncating any negative eigenvalues of this matrix to zero 15, 16.

The main difficulty that is common to all these multivariate methods is that they require the knowledge of the within‐study correlations, which are often not reported and are difficult to obtain even on request 18, 19. In other situations, the calculation of the within‐study correlations may not be easy and sometimes requires more computationally intensive methods 20. Such a challenge is emphasized by the review paper by Jackson *et al.*, 2, ‘ … perhaps the greatest difficulty applying the multivariate meta‐analysis model in practice is that the within‐study correlations are required by the model and are typically unknown’. In order to avoid the difficulty of unknown within‐study correlations, Riley *et al.* (2008) 21 proposed a novel model using a single correlation parameter to describe the total marginal correlation between outcomes. However, only likelihood based methods have been developed for fitting this model so it too can suffer from convergence problems. Wei and Higgins 22 proposed a different strategy by estimating the within‐study covariances based on information about likely correlations between underlying binary or continuous outcomes. Sensitivity analyses can also be performed with respect to the plausible correlations. In addition to these methods, other strategies have been considered, such as borrowing within‐study correlations from studies with individual participant data 5, 23, assuming plausible values for unknown correlation coefficients 7, 10 and using Bayesian framework with noninformative priors on ranges of correlation coefficients 24. However, none of these methods entirely resolve the common practical difficulty that the within‐study correlations are unknown.

The goal of this paper is to propose a simple and non‐iterative method, which avoids all the aforementioned difficulties. We propose to simply use standard methods for univariate meta‐analysis to make marginal inferences for each outcome. However, we augment the conventional separate univariate meta‐analyses by also estimating the covariances of the univariate pooled estimates. Our strategy is, therefore, very similar to the type of approach that meta‐analysts will already be familiar with and does not need the often unknown within‐study correlations. The proposed method does not suffer from any convergence difficulties and provides valid inference for joint inferences and for functions of correlated effects.

As meta‐analyses conventionally favor simple and robust procedures, the proposed method is expected to be widely applicable to practical studies. By using univariate methods for meta‐analysis to make marginal inferences for the outcomes, our procedure does not make an attempt to allow any borrowing of strength. Borrowing of strength refers to the potential for multivariate meta‐analyses to provide more precise point estimates than multiple univariate meta‐analyses of the same outcome data 2. The borrowing of strength afforded by multivariate meta‐analysis has in any case often been found to be small 25, 26. Perhaps most importantly, our proposal allows the consumers of systematic reviews that contain many univariate meta‐analyses to make appropriate joint inferences and inferences about functions of the correlated pooled estimates that may be of interest. We emphasise this use of our methodology through our second example, which comes from the Cochrane database.

This paper is organized as follows. In Section 2, we describe the standard ML and REML inferences for multivariate meta‐analysis and the method proposed by Jackson *et al.*, 15. In Section 3, we describe the proposed non‐iterative method. In Section 4, we conduct simulation studies to compare the proposed method with the existing methods and investigate the bias, coverage probability and relative efficiency. We apply the proposed method to two meta‐analyses in Section 5. Finally, we provide a brief discussion in Section 6. In addition, we provide the R code for implementing the proposed method in Appendix.

## 2 Methods for multivariate meta‐analysis

In this section, we will briefly review the methods for multivariate meta‐analysis that have recently been proposed to make valid inference for correlated outcome data in meta‐analysis.

### 2.1 Bivariate random‐effects meta‐analysis model

To simplify our presentation, we will describe the,ultivariate meta‐analysis methods for bivariate outcomes, while acknowledging that these methods can be easily extended to situations with more than two outcomes. We consider a meta‐analysis with *m* studies and two outcomes of interest. For the *i*th study, denote *Y*_*ij*_ and *s*_*ij*_ as the summary measure for the *j*th outcome of interest and the associated within‐study standard error respectively, *i* = 1,…,*m* and *j* = 1,2. Each summary measure *Y*_*ij*_ is an estimate of the true underlying study specific effect size *θ*_*ij*_. To account for heterogeneity in the underlying effect sizes across studies, we assume *θ*_*ij*_ to be independently drawn from a common distribution with overall means *β*_*j*_, between study variations $\tau_{j}^{2}$, *j* = 1,2 and a between‐study correlation *ρ*_B_.

Under the conventional normal distribution assumptions for *Y*_*ij*_ and *θ*_*ij*_, the general bivariate random‐effects meta‐analysis (BRMA) model can be written hierarchically as

(1) $$\begin{array}{cc} \left(\begin{array}{c} Y_{i1}\\ Y_{i2} \end{array}\right) & ∼\text{MVN}\left(\left(\begin{array}{c} \theta_{i1}\\ \theta_{i2} \end{array}\right),\Delta_{i}\right),\;\;\Delta_{i}=\left(\begin{array}{cc} s_{i1}^{2} & s_{i1}s_{i2}\rho_{w_{i}}\\ s_{i2}^{2} \end{array}\right),\\ \left(\begin{array}{c} \theta_{i1}\\ \theta_{i2} \end{array}\right) & ∼\text{MVN}\left(\left(\begin{array}{c} \beta_{1}\\ \beta_{2} \end{array}\right),\Omega \right),\;\;\;\;\;\Omega =\left(\begin{array}{cc} \tau_{1}^{2} & \tau_{1}\tau_{2}\rho_{B}\\ \tau_{2}^{2} \end{array}\right), \end{array}$$

where ***Δ***_*i*_ and ***Ω*** are the within‐study and between‐study covariance matrices respectively and $\rho_{w_{i}}$ and *ρ*_B_ are the within‐study correlations and between‐study correlation, respectively. The BRMA usually aims at estimating *β*_1_ and *β*_2_. Functions of the pooled estimates (e.g. ${\hat{\beta }}_{1}-{\hat{\beta }}_{2}$ or ${\hat{\beta }}_{1}/{\hat{\beta }}_{2}$) are then also often of inferential interest 9, 21.

We follow the usual convention in meta‐analysis of treating all *s*_*ij*_ as fixed and known. When the within‐study correlations $\rho_{w_{i}}$ are also known, inference for the overall effect sizes *β*_1_ and *β*_2_ are based on the marginal distribution of (*Y*_*i*1_,*Y*_*i*2_).

(2) $$\left(\begin{array}{c} Y_{i1}\\ Y_{i2} \end{array}\right)∼\text{MVN}\left(\left(\begin{array}{c} \beta_{1}\\ \beta_{2} \end{array}\right),{\text{V}}_{i}\right),\;\text{where}\;{\text{V}}_{i}=\Delta_{i}+\Omega =\left(\begin{array}{cc} s_{i1}^{2}+\tau_{1}^{2} & s_{i1}s_{i2}\rho_{\mathrm{wi}}+\tau_{1}\tau_{2}\rho_{B}\\ s_{i2}^{2}+\tau_{2}^{2} \end{array}\right).$$

We note that the variance of *Y*_*ij*_ is partitioned into two parts, $s_{\mathrm{ij}}^{2}$ and $\tau_{j}^{2}$, as in the analysis of variance for univariate random effects model, and the covariance between *Y*_*i*1_ and *Y*_*i*2_, cov(*Y*_*i*1_,*Y*_*i*2_) = *s*_*i*1_ *s*_*i*2_ *ρ*_*wi*_+*τ*_1_ *τ*_2_ *ρ*_B_, is also partitioned into two parts as the sum of within‐study and between‐study covariances. ML estimation or REML estimation can be used to make inference on the model parameters.

Given the between‐study covariance matrix ***Ω***, the best linear unbiased estimator (BLUE) of the overall effect sizes *β*_1_ and *β*_2_ can be obtained through weighted least square estimation

(3) $$\hat{\beta }=\left(\begin{array}{c} {\hat{\beta }}_{1}\\ {\hat{\beta }}_{2} \end{array}\right)={\left\{\overset{m}{\underset{i=1}{\sum }}{\left(\Omega +\Delta_{i}\right)}^{-1}\right\}}^{-1}\left\{\overset{m}{\underset{i=1}{\sum }}{\left(\Omega +\Delta_{i}\right)}^{-1}\left(\begin{array}{c} Y_{i1}\\ Y_{i2} \end{array}\right)\right\},$$

and the estimators $\left({\hat{\beta }}_{1},{\hat{\beta }}_{2}\right)$ are approximately normally distributed with covariance matrix

(4) $$\hat{\Sigma }={\left\{\overset{m}{\underset{i=1}{\sum }}{\left(\Omega +\Delta_{i}\right)}^{-1}\right\}}^{-1}.$$

However, the between‐study covariance matrix ***Ω*** must be estimated in practice. ML estimation of all parameters can be performed but maximum likelihood estimates of variance components such as ***Ω*** are, in general, biased downwards. REML estimation of ***Ω*** has been proposed in order to reduce this bias and is the default procedure for ‘mvmeta’ packages in R 27 and Stata 28. The between‐study covariance ***Ω*** is estimated using REML by maximizing the restricted likelihood

$$-\frac{1}{2}\left[\log\left(\left|\overset{m}{\underset{i=1}{\sum }}{\left(\Omega +\Delta_{i}\right)}^{-1}\right|\right)+\overset{m}{\underset{i=1}{\sum }}\left\{\log|\Omega +\Delta_{i}|+{\left({\text{Y}}_{i}-\hat{\beta }\right)}^{T}{\left(\Omega +\Delta_{i}\right)}^{-1}({\text{Y}}_{i}-\hat{\beta })\right\}\right].$$

Once the between‐study covariance matrix has been estimated, the standard procedure for performing multivariate meta‐analyses 2 replaces ***Ω*** with $\hat{\Omega }$ in (3) and (4). We denote the estimates using the REML estimator of ***Ω*** as $\left({\hat{\beta }}_{1R},{\hat{\beta }}_{2R}\right)$.

### 2.2 Jackson's method of moments

Meta‐analysis methods have been intrinsically in favor of simple and robust procedures. For example, the non‐iterative method for univariate meta‐analysis by DerSimonian and Laird 1 has been cited more than 13 000 times to date according to Google Scholar. The case for preferring simple and robust methods is perhaps even stronger in the multivariate case because likelihood based estimation of the variance components becomes computationally challenging in high dimensions and multivariate meta‐analyses make stronger assumptions than univariate meta‐analyses 2.

An alternative non‐iterative method of moments for fitting the BRMA has been proposed by Jackson *et al.*, 15. This is a natural and easily implemented multivariate extension of DerSimonian and Laird's method (hereafter referred to as Jackson's method). Specifically, to incorporate the situation where some studies only report one of the two outcomes, they denoted $R_{Y_{j}}$ as the set of studies reporting *Y*_*j*_ and $R_{Y_{1},Y_{2}}$ as the set of studies reporting both *Y*_1_ and *Y*_2_. Jackson *et al.* proposed the following multivariate **Q** statistic (or heterogeneity statistic)

(5) $$\text{Q}=\left[\begin{array}{cc} Q_{1} & Q_{12}\\ Q_{2} \end{array}\right]=\left[\begin{array}{cc} \underset{i\in R_{Y_{1}}}{\sum }\frac{{\left(Y_{i1}-{\bar{Y}}_{1}\right)}^{2}}{s_{i1}^{2}} & \underset{i\in R_{Y_{1},Y_{2}}}{\sum }\frac{\left(Y_{i1}-{\bar{Y}}_{1}^{*}\right)\left(Y_{i2}-{\bar{Y}}_{2}^{*}\right)}{s_{i1}s_{i2}}\\ \underset{i\in R_{Y_{2}}}{\sum }\frac{{\left(Y_{i2}-{\bar{Y}}_{2}\right)}^{2}}{s_{i2}^{2}} \end{array}\right],$$

where ${\bar{Y}}_{j}$ denotes the weighted mean of *Y*_*j*_ over studies reporting *Y*_*j*_ with weights $1/s_{i1}^{2}$, and ${\bar{Y}}_{j}^{*}$ denotes the weighted mean of *Y*_*j*_ over studies reporting both *Y*_1_ and *Y*_2_ with weights 1/*s*_*i*1_ *s*_*i*2_ (*j* = 1,2),

$${\bar{Y}}_{1}=\frac{\underset{i\in R_{Y_{1}}}{\sum }Y_{i1}/s_{i1}^{2}}{\underset{i\in R_{Y_{1}}}{\sum }1/s_{i1}^{2}},\;{\bar{Y}}_{1}^{*}=\frac{\underset{i\in R_{Y_{1},Y_{2}}}{\sum }Y_{i1}/(s_{i1}s_{i2})}{\underset{i\in R_{Y_{1},Y_{2}}}{\sum }1/(s_{i1}s_{i2})},\;{\bar{Y}}_{2}=\frac{\underset{i\in R_{Y_{2}}}{\sum }Y_{i2}/s_{i2}^{2}}{\underset{i\in R_{Y_{2}}}{\sum }1/s_{i2}^{2}}\;\text{and}\;{\bar{Y}}_{2}^{*}=\frac{\underset{i\in R_{Y_{1},Y_{2}}}{\sum }Y_{i2}/(s_{i1}s_{i2})}{\underset{i\in R_{Y_{1},Y_{2}}}{\sum }1/(s_{i1}s_{i2})}.$$

By equating the entries of the **Q** statistic with their expectations, Jackson *et al.*, 15 solved the following linear equations for parameters in the between‐study covariance matrix ***Ω***, namely the between‐study heterogeneity $\tau_{1}^{2}$ and $\tau_{2}^{2}$ and the between‐study covariance *ρ*_B_ *τ*_1_ *τ*_2_,

(6) $$\left\{\begin{array}{c} Q_{1}=E[Q_{1}]=(m_{Y_{1}}-1)+\left(\underset{i\in R_{Y_{1}}}{\sum }s_{i1}^{-2}-\underset{i\in R_{Y_{1}}}{\sum }s_{i1}^{-4}/\underset{i\in R_{Y_{1}}}{\sum }s_{i1}^{-2}\right)\tau_{1}^{2}\\ Q_{2}=E[Q_{2}]=(m_{Y_{2}}-1)+\left(\underset{i\in R_{Y_{2}}}{\sum }s_{i2}^{-2}-\underset{i\in R_{Y_{2}}}{\sum }s_{i2}^{-4}/\underset{i\in R_{Y_{2}}}{\sum }s_{i2}^{-2}\right)\tau_{2}^{2}\\ Q_{12}=E[Q_{12}]=a+b\rho_{B}\tau_{1}\tau_{2} \end{array}\right.$$

where $m_{Y_{j}}$ is the number of studies with outcome *Y*_*j*_ (*j* = 1,2),

(7) $$a=\left\{\underset{i\in R_{Y_{1},Y_{2}}}{\sum }\rho_{w_{i}}\right\}-\frac{\underset{i\in R_{Y_{1},Y_{2}}}{\sum }\rho_{\mathrm{wi}}/(s_{i1}s_{i2})}{\underset{i\in R_{Y_{1},Y_{2}}}{\sum }1/(s_{i1}s_{i2})}\;\text{and}\;b=\left\{\underset{i\in R_{Y_{1},Y_{2}}}{\sum }\frac{1}{s_{i1}s_{i2}}\right\}-\frac{\underset{i\in R_{Y_{1},Y_{2}}}{\sum }1/\left(s_{i1}^{2}s_{i2}^{2}\right)}{\underset{i\in R_{Y_{1},Y_{2}}}{\sum }1/(s_{i1}s_{i2})}.$$

Denote ${\hat{\tau }}_{1J}^{2}$, ${\hat{\tau }}_{2J}^{2}$ and ${\hat{\rho }}_{\mathrm{BJ}}^{2}$ the solutions of Equation (6), and ${\hat{\Omega }}_{J}$ the corresponding estimated between‐study covariance matrix after truncation, where required, to ensure that ${\hat{\Omega }}_{J}$ is positive semi‐definite. It is worth mentioning that, in the bivariate case, the truncation of negative eigenvalues is a way to truncate any negative estimated between‐study variance to zero and also to truncate any estimated between‐study correlation to (−1,1) inclusively. The estimators of overall effect sizes $\left({\hat{\beta }}_{1J},{\hat{\beta }}_{2J}\right)$ and their covariance matrix take the same form as Equations (3) and (4) with ***Ω*** replaced by ${\hat{\Omega }}_{J}$.

The estimated between‐study variances ${\hat{\tau }}_{1J}^{2}$ and ${\hat{\tau }}_{2J}^{2}$ are the same as the estimates from Dersimonian and Laird's univariate meta‐analysis method. An advantage of Jackson's method is that the non‐iterative estimation procedure avoids the convergence problems that can be encountered in ML and REML estimation. Jackson's method addresses the singular covariance matrix problem by constructing a ‘truncated’ symmetric and positive semi‐definite estimate of ***Ω*** by truncating any negative eigenvalues to zero; for details, refer to Jackson *et al.*, 15. Finally, under missing complete at random assumption, for studies with missing outcomes, a large within‐study standard error, zero outcomes and zero within‐study correlations can be assigned to the missing effect sizes so that Equation (3) can be used to conveniently estimate the overall effect sizes *β*_1_ and *β*_2_ 15. For further details of the computational problems that can arise when fitting the BRMA model, see Hamza *et al.*, 14.

## 3 The proposed marginal method of moments

In this section, we will develop our proposal. Before truncation, Jackson's method uses the univariate estimates of the between‐study variance in the multivariate meta‐analysis. Our proposal also does this and goes further by also using the univariate point estimates of the overall effect sizes.

### 3.1 Estimation

As we discussed in the introduction, one difficulty when using any method for fitting the BRMA model is that the within‐study correlations $\rho_{w_{i}}$ (*i* = 1,…,*m*) are often unknown 2, 21. Because the within‐study correlations are involved in model (2), the methods described previously in Section 2 are not immediately applicable in situations when the within‐study correlations are unknown.

To avoid this issue, we propose a simple and non‐iterative method. Our method is conservative as it allows no borrowing of strength, but it allows further inferences to be made that the usual univariate methods do not allow. Our argument is that, without the within‐study correlations, it is not clear how much borrowing of strength is possible or appropriate, and so, we do not permit any. The strategy is very simple: we use conventional univariate meta‐analysis results for the marginal inferences for each outcome, but we further estimate the covariances between these univariate point estimates in order to make further inferences.

Specifically, we note that the univariate estimate of overall effect size *β*_*j*_ takes the form of weighted sum of *Y*_*ij*_ as

(8) $${\tilde{\beta }}_{j}=\frac{\underset{i\in R_{Y_{j}}}{\sum }w_{\mathrm{ij}}Y_{\mathrm{ij}}}{\underset{i\in R_{Y_{j}}}{\sum }w_{\mathrm{ij}}},$$

where the weights $w_{\mathrm{ij}}=1/s_{\mathrm{ij}}^{2}$ if a fixed effect model is adopted and $w_{\mathrm{ij}}={\left(s_{\mathrm{ij}}^{2}+{{\tilde{\tau }}_{j}}^{2}\right)}^{-1}$ if a random effects model is adopted, where ${\tilde{\tau }}_{j}^{2}=\max\left\{0,\frac{Q_{j}-(m_{Y_{j}}-1)}{\underset{i\in R_{Y_{j}}}{\sum }s_{\mathrm{ij}}^{-2}-\underset{i\in R_{Y_{j}}}{\sum }s_{\mathrm{ij}}^{-4}/\underset{i\in R_{Y_{j}}}{\sum }s_{\mathrm{ij}}^{-2}}\right\}$ and *Q*_*j*_ is defined in Equation (5). Alternative estimators of $\tau_{j}^{2}$ could also be used in (8). Marginal inference then proceeds using a normal approximation for ${\tilde{\beta }}_{j}$ where

$${\tilde{\beta }}_{j}∼\text{MVN}\left(\beta_{j},\;\;{\left\{\underset{i\in R_{Y_{j}}}{\sum }w_{\mathrm{ij}}\right\}}^{-1}\right)$$

so that the usual univariate results are recovered for *β*_*j*_. This is an important advantage of our proposal: we regain the univariate results that meta‐analysts will already be familiar with for each of the estimated effects. Our methodology therefore recovers all the results from all the very many systematic reviews conducted to date that use univariate meta‐analysis techniques.

In situations where we wish to further perform joint inference for (*β*_1_,*β*_2_) or to make inferences about a function of the form *f*(*β*_1_,*β*_2_), we must take into account the correlation between estimates that come from the same study. To do this, we estimate the covariance between ${\tilde{\beta }}_{1}$ and ${\tilde{\beta }}_{2}$ as

(9) $$\text{cov}({\tilde{\beta }}_{1},{\tilde{\beta }}_{2})=\underset{i\in R_{Y_{1},Y_{2}}}{\sum }\frac{w_{i1}}{w_{1+}}\frac{w_{i2}}{w_{2+}}\text{cov}(Y_{i1},Y_{i2})\approx \underset{i\in R_{Y_{1},Y_{2}}}{\sum }\frac{w_{i1}}{w_{1+}}\frac{w_{i2}}{w_{2+}}(Y_{i1}-{\tilde{\beta }}_{1})(Y_{i2}-{\tilde{\beta }}_{2}),$$

where *w*_*j*+_ is the sum of weights *w*_*ij*_ for outcome *Y*_*j*_ over the studies where *Y*_*j*_ is reported, that is, $w_{j+}=\underset{i\in R_{Y_{j}}}{\sum }w_{\mathrm{ij}}$. We note that in the last step of Equation (9), we approximate cov(*Y*_*i*1_,*Y*_*i*2_) by its empirical counterpart $\left(Y_{i1}-{\tilde{\beta }}_{1})(Y_{i2}-{\tilde{\beta }}_{2}\right)$. This is a similar approach to the one suggested by Hedges *et al.*, 29. If no study provides estimates of both *β*_1_ and *β*_2_, then we obtain an empty sum in (9) so that $\text{cov}({\tilde{\beta }}_{1},{\tilde{\beta }}_{2})=0$. This is appropriate because if disjoint sets of studies provide estimates of *β*_1_ and *β*_2_ then the pooled estimates are independent.

We can therefore take the estimators ${\tilde{\beta }}_{1}$ and ${\tilde{\beta }}_{2}$ as approximately normally distributed, centred at *β*_1_ and *β*_2_, with covariance matrix

(10) $${\tilde{\Sigma }}^{*}=\left(\begin{array}{cc} {\left(\underset{i\in R_{Y_{1}}}{\sum }w_{i1}\right)}^{-1} & \underset{i\in R_{Y_{1},Y_{2}}}{\sum }\frac{w_{i1}}{w_{1+}}\frac{w_{i2}}{w_{2+}}(Y_{i1}-{\tilde{\beta }}_{1})(Y_{i2}-{\tilde{\beta }}_{2})\\ {\left(\underset{i\in R_{Y_{2}}}{\sum }w_{i2}\right)}^{-1} \end{array}\right).$$

The proposed procedure is different to conducting two separate univariate meta‐analysis using outcome data on *Y*_1_ and *Y*_2_ because it accounts for the correlations between the outcomes *Y*_1_ and *Y*_2_ through the off‐diagonal element in ${\tilde{\Sigma }}^{*}$ when making joint inferences about (*β*_1_,*β*_2_) or functions of these parameters. If the matrix ${\tilde{\Sigma }}^{*}$ is not positive semi‐definite, then we truncate any negative eigenvalues to zero in the way proposed by Jackson *et al.*, 15 for the estimated between‐study covariance matrix 15. We hereby refer to the proposed method as the marginal method of moments (MMoM) because we obtain the usual univariate marginal results when using this method. An important difference between the MMoM and Jackson's method is that the within‐study correlations $\rho_{w_{i}}$ are not required in the proposed method (Equations (8),  (9) and (10)) but are needed by Jackson's method (Equations (3) and (4)). The proposed marginal method of moments is implemented in the R software package ‘xmeta’, which is freely distributed under GNU General Public License (GPL) and can directly be installed from CRAN (http://cran.r‐project.org/package=xmeta/), the official R package archive.

### 3.2 Functions of the estimated overall effect sizes

If the goal is to make inferences about linear functions of the effect sizes (e.g. *β*_1_−*β*_2_), then the distribution of any linear combination of ${\tilde{\beta }}_{1}$ and ${\tilde{\beta }}_{2}$ can be obtained from the approximation in the previous subsection and used to make inferences. If the goal is to make inferences about a non‐linear function (e.g. *β*_1_/*β*_2_), then the delta method can be used as a further approximation. Therefore, the MMoM provides a non‐iterative procedure to obtain valid inference for any function of the effect sizes whilst correctly accounting for the correlation among estimated effect sizes.

### 3.3 Missing outcome data

In practice, it is common that only a proportion of studies have all outcomes reported, and the remaining studies have some of outcomes missing. Our methodology explains how to analyse datasets with missing outcome data but, in this subsection, we discuss the implications of any missing data.

Our approach is a non‐likelihood‐based classical method, and so, we require the missing completely at random (MCAR) assumption when encountering missing data and using the MMoM 30. Although this assumption may not be true in some applications, it is in any case instructive to consider MCAR as a step toward missing at random (MAR) and then missing not at random (MNAR) modelling. Extensions of the proposed method are, however, required to justify the weaker assumptions of MAR and MNAR, and we return to this issue in the discussion.

Under MCAR, the computation with missing data can be conveniently performed using complete data methods upon allocating very large within‐study variances (e.g. 10^6^) to the missing observations, where the missing study outcomes and the corresponding within‐study correlations are set to zero. A proof of the equivalence between this computationally convenient approach and the formula in Section 3.1 are provided in the Supporting Information.

### 3.4 Extension to meta‐regression

In many applications, study‐level covariates are available, such as mean age, percentage female, and year of publication. These covariates may be incorporated in the meta‐analysis in order to explain some of the between‐study variation. In this subsection, we explain how we can extend our method to the meta‐regression setting. We assume the estimate of outcome *j* in the *i*‐th study *Y*_*ij*_ has mean of ${\text{X}}_{\mathrm{ij}}^{T}\beta_{j}$ and variance $\sigma_{\mathrm{ij}}^{2}+\tau_{j}^{2}$ for *i* = 1,…,*m* and *j* = 1,2, where ***X***_*ij*_ denotes for the *p*_*j*_×1 covariates vector that may correlate with the outcome *Y*_*ij*_ and ***β***_*j*_ is the *p*_*j*_×1 vector of regression coefficients.

The heterogeneity meta‐regression **Q** statistic for outcome *Y*_*j*_, *Q*_*j*_, can be calculated as

$$Q_{j}=\underset{i\in R_{Y_{j}}}{\sum }\sigma_{\mathrm{ij}}^{-2}{\left(Y_{\mathrm{ij}}-{\text{X}}_{\mathrm{ij}}^{T}{\hat{\beta }}_{j,\text{fix}}\right)}^{2},\;\text{for}\;j=1,2,$$

where ${\hat{\beta }}_{j,\text{fix}}={\left({\text{X}}_{j}^{T}\Lambda_{j}^{-1}{\text{X}}_{j}\right)}^{-1}{\text{X}}_{j}^{T}\Lambda_{j}^{-1}{\text{Y}}_{j}$ denotes the maximum likelihood estimate of *β*_*j*_ under the fixed effects assumption that $\tau_{j}^{2}=0$, **X**_*j*_ is the *p*_*j*_×*m* design matrix, **Y**_*j*_=(*Y*_1*j*_,...,*Y*_*mj*_)^*T*^ is the vector for outcome *j* and $\Lambda_{j}=\text{diag}\left(\sigma_{1j}^{2},\ldots ,\sigma_{\mathrm{mj}}^{2}\right)$ is the diagonal covariance matrix with the within‐study variances of each element of **Y**_*j*_ as the diagonal elements.

By equating the empirical moments with their expectations, we can obtain the estimates of between‐study variance $\tau_{j}^{2}$ by solving

(11) $$Q_{j}=E[Q_{j}]=(m-p_{j})+\left[\text{tr}\left(\Lambda_{j}^{-1}\right)-\text{tr}\left\{{\left({\text{X}}_{j}^{T}\Lambda_{j}^{-1}{\text{X}}_{j}\right)}^{-1}{\text{X}}_{j}^{T}\Lambda_{j}^{-2}{\text{X}}_{j}\right\}\right]\tau_{j}^{2}.$$

Solving Equation (11) yields the DerSimonian and Laird moment estimator

$$\tau_{j}^{2}=\max\left[0,\frac{Q_{j}-(m-p_{j})}{\text{tr}\left(\Lambda_{j}^{-1}\right)-\text{tr}\left\{{\left({\text{X}}_{j}^{T}\Lambda_{j}^{-1}{\text{X}}_{j}\right)}^{-1}{\text{X}}_{j}^{T}\Lambda_{j}^{-2}{\text{X}}_{j}\right\}}\right]\;\text{for}\;j=1,2.$$

The estimate of the overall treatments effect are given by ${\tilde{\beta }}_{j}={\left({\text{X}}_{j}^{T}{\Lambda_{j}^{*}}^{-1}X_{j}\right)}^{-1}X_{j}^{T}{\Lambda_{j}^{*}}^{-1}Y_{j}$, where $\Lambda_{j}^{*}=\text{diag}\left(\sigma_{1j}^{2}+{\hat{\tau }}_{j}^{2},\ldots ,\sigma_{\mathrm{mj}}^{2}+{\hat{\tau }}_{j}^{2}\right)$ denotes the diagonal covariance matrix under the random effects assumption with the sum of the within‐study and between‐study variances as the diagonal elements.

The meta‐regression extension of the proposed MMoM can account for any correlation between the outcomes. It can be shown that $\left({\tilde{\beta }}_{1},{\tilde{\beta }}_{2}\right)$ is approximately normally distributed with the mean (*β*_1_,*β*_2_) and the covariance matrix

$$\begin{array}{c} {\tilde{\Sigma }}^{*}={\left(\;\;\begin{array}{cc} {\left(X_{1}^{T}{\Lambda_{1}^{*}}^{-1}X_{1}\right)}_{p_{1}\times p_{1}}^{-1} & \;{\left(X_{1}^{T}{\Lambda_{1}^{*}}^{-1}X_{1}\right)}^{-1}X_{1}^{T}{\Lambda_{1}^{*}}^{-1}\text{cov}\left(Y_{1},Y_{2}\right){\Lambda_{2}^{*}}^{-1}X_{2}{\left(X_{2}^{T}{\Lambda_{2}^{*}}^{-1}X_{2}\right)}_{p_{1}\times p_{2}}^{-1}\\ {\left(X_{2}^{T}{\Lambda_{2}^{*}}^{-1}X_{2}\right)}_{p_{2}\times p_{2}}^{-1} \end{array}\;\;\right)}_{\left(p_{1}+p_{2})\times (p_{1}+p_{2}\right)}, \end{array}$$

where cov(***Y***_1_,***Y***_2_) is approximately estimated by

$$\text{diag}\left\{\left(Y_{11}-X_{11}^{T}{\hat{\beta }}_{1,\text{fix}}\right)\left(Y_{12}-X_{12}^{T}{\hat{\beta }}_{2,\text{fix}}\right),\ldots ,\left(Y_{m1}-X_{m1}^{T}{\hat{\beta }}_{1,\text{fix}}\right)\left(Y_{m2}-X_{m2}^{T}{\hat{\beta }}_{2,\text{fix}}\right)\right\},$$

which is a diagonal matrix with $\left(Y_{i1}-X_{i1}^{T}{\hat{\beta }}_{1,\text{fix}}\right)\left(Y_{i2}-X_{i2}^{T}{\hat{\beta }}_{2,\text{fix}}\right)$ being the *i*‐th diagonal element. Again, the eigenvalues of ${\tilde{\Sigma }}^{*}$ can be truncated to ensure this matrix is positive semi‐definite and the methods and issues for handling missing data described in Section 3.3 apply. This extension for meta‐regression reduces to the MMoM for meta‐analysis if there are no covariates and an intercept only ‘regression’ is used.

## 4 Simulation study

To evaluate the finite sample performance of the proposed MMoM and to compare it with some more established multivariate methods, in this section, we will conduct a simulation study. Here, data are generated from a two‐stage procedure as specified in Equation (1). To cover a wide spectrum of scenarios, we vary the values for the four factors that are considered important in practice.

Specifically, the number of studies *m* is set to either 10 or 25 to represent meta‐analyses with moderate number and large numbers of studies, respectively. We will consider both complete data and missing data scenarios. For the missing data scenario, there are 30*%* missing data for each of the two outcomes, where data are MCAR. To reflect the variation in the within‐study standard errors, we sample $s_{\mathrm{ij}}^{2}$ as the square of an *N*(0.25,0.50) distribution, which leads to a median value of around 0.26. The size of the within‐study variation relative to the between‐study variation may have a substantial impact on the performance of the methods. To this end, we let the between‐study variation to be relatively small, comparable or relatively large, corresponding to $\tau_{1}^{2}=\tau_{2}^{2}=0.1$, 0.25 or 0.5 respectively. For between‐study correlations, we set *ρ*_B_ to be either −0.8, −0.6, −0.4, 0, 0.4, 0.6 or 0.8. Finally, for the within‐study correlations, we set $\rho_{w_{i}}$ for all studies to either −0.8, −0.5, 0, 0.5 or 0.8.

We set the overall effect sizes to be *β*_1_=0 and *β*_2_=2. The novel aspect of our method is that it is intended to make valid joint inferences and inferences for functions of *β*_1_ and *β*_2_. We assume that the target for inference is the difference between the effect sizes of two outcomes, that is, *δ* = *β*_1_−*β*_2_. The parameter *δ* is estimated as ${\tilde{\beta }}_{1}-{\tilde{\beta }}_{2}$ using the MMoM and as ${\hat{\beta }}_{1R}-{\hat{\beta }}_{2R}$ and ${\hat{\beta }}_{1J}-{\hat{\beta }}_{2J}$ using BRMA, where the between‐study covariance is estimated by REML and Jackson's method, respectively. The standard errors of the estimates of *δ* are calculated as (1,−1)^*T*^V(1,−1), where V is the corresponding covariance matrix of the estimates of (*β*_1_,*β*_2_). For each simulation setting, we generated 1000 samples. The samples were simulated in R (R Development Core Team, Version 3.14.1) using the ‘mvrnorm’ function.

**Figure 1:**
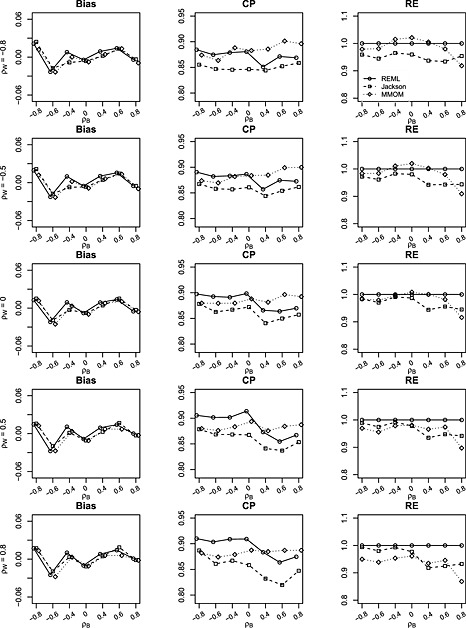
Bias, coverage probability (CP) and relative efficiency (RE) of estimates for *δ* = *β*_1_−*β*_2_ from restricted maximum likelihood (REML), Jackson's method (Jackson) and marginal method of moments (MMoM) based on 1000 simulations with data generated from bivariate random‐effects meta‐analysis model. The between‐study/within‐study variation ratio is close to 2 (i.e. between‐study variations $\tau_{1}^{2}=\tau_{2}^{2}=0.5$ and median within‐study variation is 0.26). Within‐study correlations *ρ*_*w*_ are set to (−0.8,−0.5,0,0.5,0.8). Between‐study correlations *ρ*_*b*_ are set to (−0.8,−0.6,−0.4,0,0.4,0.6,0.8). Number of studies is *m* = 10. There is no missing data.

**Figure 2:**
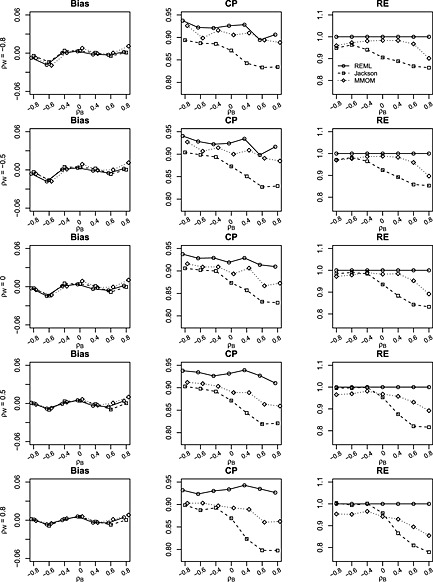
Bias, coverage probability (CP) and relative efficiency (RE) of estimates for *δ* = *β*_1_−*β*_2_ from restricted maximum likelihood (REML), Jackson's method (Jackson) and marginal method of moments (MMoM) based on 1000 simulations with data generated from bivariate random‐effects meta‐analysis model. The between‐study/within‐study variation ratio is close to 2 (i.e. between‐study variations $\tau_{1}^{2}=\tau_{2}^{2}=0.5$ and median within‐study variation is 0.26). Within‐study correlations *ρ*_*w*_ are set to (−0.8,−0.5,0,0.5,0.8). Between‐study correlations *ρ*_*b*_ are set to (−0.8,−0.6,−0.4,0,0.4,0.6,0.8). Number of studies is *m* = 25. There is no missing data.

**Figure 3:**
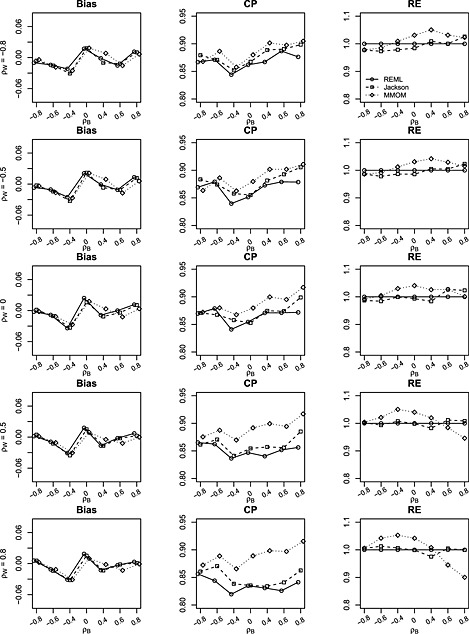
Bias, coverage probability (CP) and relative efficiency (RE) of estimates for *δ* = *β*_1_−*β*_2_ from restricted maximum likelihood (REML), Jackson's method (Jackson) and marginal method of moments (MMoM) based on 1000 simulations with data generated from bivariate random‐effects meta‐analysis model. The between‐study/within‐study variation ratio is close to 2 (i.e. between‐study variations $\tau_{1}^{2}=\tau_{2}^{2}=0.5$ and median within‐study variation is 0.26). Within‐study correlations *ρ*_*w*_ are set to (−0.8,−0.5,0,0.5,0.8). Between‐study correlations *ρ*_*b*_ are set to (−0.8,−0.6,−0.4,0,0.4,0.6,0.8). Number of studies is *m* = 10. There are 30*%* missing data for each of the two outcomes.

**Figure 4:**
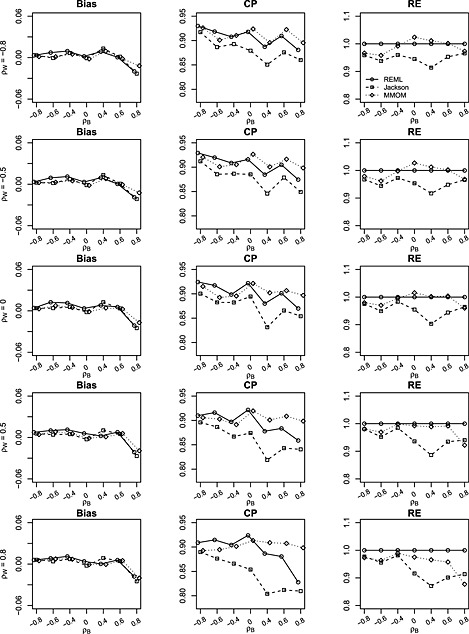
Bias, coverage probability (CP) and relative efficiency (RE) of estimates for *δ* = *β*_1_−*β*_2_ from restricted maximum likelihood (REML), Jackson's method (Jackson) and marginal method of moments (MMoM) based on 1000 simulations with data generated from bivariate random‐effects meta‐analysis model. The between‐study/within‐study variation ratio is close to 2 (i.e. between‐study variations $\tau_{1}^{2}=\tau_{2}^{2}=0.5$ and median within‐study variation is 0.26). Within‐study correlations *ρ*_*w*_ are set to (−0.8,−0.5,0,0.5,0.8). Between‐study correlations *ρ*_*b*_ are set to (−0.8,−0.6,−0.4,0,0.4,0.6,0.8). Number of studies is *m* = 25. There are 30*%* missing data for each of the two outcomes.

The results from the BRMA model, fitted using both REML and Jackson's method, were produced in order to compare the MMoM results with BRMA. However, we note that the BRMA model makes use of further data (the within‐study correlations), and the data were simulated under the BRMA model, so we cannot anticipate that our method will perform better than the alternatives. Instead, our main interest is whether or not our much simpler proposal performs similarly to BRMA. We present the results for $\tau_{1}^{2}=\tau_{2}^{2}=0.5$ in Figures 1, 2, 3, 4; the results for $\tau_{1}^{2}=\tau_{2}^{2}=0.25$ and 0.1 are provided in Section 3 of the Supporting Information. We show the results for the largest between‐study variance in the main paper because we anticipate that considerable between‐study heterogeneity would present the biggest challenge to our method. However, the overall conclusions are quite similar for all three between‐study variances (see Section 3 of the Supporting Information). As the between‐study variation becomes smaller (i.e. $\tau_{1}^{2}=\tau_{2}^{2}=0.25$ or 0.1), all methods generally provide better (closer to the nominal) actual coverage probabilities.

Figure 1 summarizes the empirical bias (Bias), the coverage probability (CP) of nominal 95*%* confidence intervals and relative efficiency (RE) of the estimate of *δ* = *β*_1_−*β*_2_ estimated using BRMA (REML), BRMA (Jackson) and the MMoM when there are no missing data (referred to as the complete data setting) and there are *m* = 10 studies. The total number of simulated data sets (1000) is used to calculate CP of Jackson's method and MMoM. RE is defined as the square of the standard error of the estimator from BRMA (REML) divided by the standard error of an estimator from a method under comparison. The left panels in Figure 1 suggest that all three methods give unbiased estimates. The middle panels indicate that confidence intervals of BRMA (REML) have slightly better coverage than confidence intervals from BRMA (Jackson). The proposed MMoM leads to similar coverage to BRMA (Jackson). Although both the MMoM and BRMA (Jackson) have the advantage of non‐iterative and computationally simple, the MMoM can be applied to the situation when the within‐study correlations are unknown. The right panels in Figure 1 present the RE of BRMA (Jackson) and the MMoM compared with the BRMA (REML). The range of RE is [92.5,99.1] for BRMA (Jackson) method and is [86.8,101.9] for the MMoM. This suggests that the MMoM is as good as Jackson's method in terms of coverage and efficiency and is only slightly worse than the iterative REML method. Interestingly, the information on within‐study correlations $\rho_{w_{i}}$ does not appear to improve the efficiency in estimating the difference in the effect sizes *δ* = *β*_1_−*β*_2_. Such a finding for complete data is in agreement with the literature 10, 25, 31. Similar observations can be found from simulation results when correlations are −0.9 or 0.9, where the CP of the proposed method is consistently better than the Jackson's method (see Figure S9−S12 of the Supporting Information).

Figure 2 presents the results when the number of studies is larger (*m* = 25). Again, there is no evidence of bias. The coverage of the MMoM is around 90*%* and is robust to the between‐study and within‐study correlations, whereas the coverage of Jackson's method deteriorates as the between‐study correlation becomes larger. The coverage of the REML method is around 93*%*, suggesting that the REML method does have advantage in coverage over Jackson's method and the MMoM when number of studies is relatively large and the within‐study correlations are available. The RE of the MMoM is ranging from 89.5*%* to 98.8*%* and is substantially better than that of Jackson's method.

Figure 3 summarizes the results when number of studies is 10 with 30*%* missing for each outcome (referred to as the missing data setting). Similar to that, in the complete data setting, there is no evidence of bias in any method. The coverages of all three methods are poorer than that in the complete data setting, but the MMoM provides at least as good coverage as the competing methods. The range of RE is [97.8,102.6] for BRMA (Jackson) and is [94.6,105.1] for the MMoM. This indicates that both Jackson's method and the proposed MMoM are as efficient as the iterative REML method in small samples despite the missing outcome data.

Figure 4 presents the results when number of studies is larger (*m* = 25) and 30*%* of data missing for each outcome. The coverages of the three methods are all improved compared with Figure 3. The coverage of the MMoM is comparable with the BRMA (REML) and is slightly better than that of BRMA (Jackson). The ranges of RE are [88.6,98.5] and [88.7,102.7] for BRMA (Jackson) and the MMoM, respectively. This suggests that the MMoM may be slightly more efficient than Jackson's method in this setting. This may be explained by the fact that the estimation of between‐study correlation *ρ*_B_ is not required by the MMoM, and truncation of the estimated between‐study covariance matrix is often performed by Jackson's method. However, it appears that BRMA (REML) may be the most efficient method in this situation, which might be anticipated because outcome data are missing and likelihood based estimation is fully efficient.

It is worth mentioning that the coverages of all three methods under comparison are less than 95% nominal size in the simulations (Figures 1–4), which is possibly due to the finite sample issue. The number of studies we used in simulation is only 10 or 25 which is relatively small. By comparing the coverage for the same method for *m* = 10 with the coverage for *m* = 25, there is a clear improvement. We also note that for all simulation settings considered, a non‐positive definite estimated covariance matrix ${\tilde{\Sigma }}^{*}$ as defined in Equation (10) was encountered in at most just 3.6*%* of simulated datasets. When a non‐positive definite covariance matrix is encountered, a positive semi‐definite version was obtained by truncating negative eigenvalues to zero as explained in Section 3.1. Estimates of the between‐study correlation in the BRMA often lie at the edge of the parameter space, which is known to result in estimation difficulties. The fact that non‐positive semi‐definite covariance matrices are such an uncommon occurrence when using the proposed method may explain why the proposed method provides quite efficient estimates of *δ* = *β*_1_−*β*_2_ despite the fact that it affords no possibility of borrowing of strength. On the other hand, the percentage of times that we encounter a singular between‐study covariance matrix using Jackson's method is at most in 56.7% of simulated datasets, in the missing data setting with *m* = 10. Because Jackson's method truncates any negative eigenvalues of the estimated between‐study covariance matrix to zero, it guarantees that the estimated between‐study covariance is always positive semi‐definite. Therefore, the estimated covariance matrices ${\hat{\Sigma }}^{*}$ from Jackson's method are positive semi‐definite in all simulated data sets.

In summary, the simulation studies suggest that under both the complete data setting and the missing data setting, the MMoM performs well in that it provides parameter estimates with small biases, retains good coverage probabilities and has high relative efficiency. Just as importantly, the MMoM can be used for meta‐analysis when within‐study correlations are unknown, which is commonly encountered in practice 2. Therefore, the proposed MMoM provides a useful and simple alternative to the existing methods.

## 5 Data applications

We illustrate the proposed MMoM by two meta‐analyses. For the first meta‐analysis, the within‐study correlations are known, and so, we compare the performance of the proposed MMoM with conventional multivariate random effects meta‐analyses. For the second meta‐analysis, the within‐study correlations are unavailable, and so, we only apply our proposed method. An additional example is shown in Section 4 of the Supporting Information.

### 5.1 Example 1: Treatment effect of antihypertensive drug on cardiovascular disease and stroke

**Figure 5:**
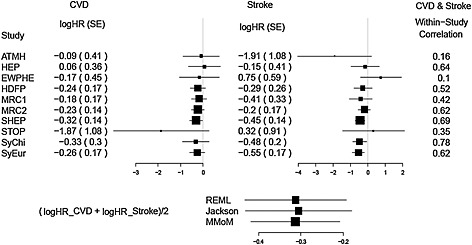
Average of log hazard ratios of cardiovascular disease (CVD) and stroke (*δ*) and 95*%* confidence intervals evaluated by the bivariate random‐effects meta‐analysis (restricted maximum likelihood (REML)), bivariate random‐effects meta‐analysis (Jackson) and the marginal method of moments (MMoM).

High blood pressure is believed as an important risk factor of heart diseases and stroke 32. Wang *et al.* performed a quantitative overview of trials to investigate the effects of lowering of systolic blood pressure (SBP) and diastolic blood pressure (DBP) on the prevention of cardiovascular disease (CVD) and stroke. They selected 10 trials in which active antihypertensive drugs were compared with placebo. A unique feature of this quantitative overview is that the individual patient data for all trials are available, which leads to the availability of within‐study correlations. Their results confirmed that antihypertensive treatment lowered SBP and DBP and reduced the risk of all cardiovascular events and stroke. The effect sizes of the antihypertensive treatment on CVD and stroke are summarized in the upper left panel of Figure 5 and are taken from Riley *et al.*, 34.

To evaluate the overall effect of the antihypertensive treatment on both CVD and stroke, it is natural to estimate the average of log hazard ratio of CVD and stroke as a composite treatment effect of the two outcomes. Such a composite effect can be used for medical decision making. Let *Y*_*i*1_ denote the log hazard ratio of risk of CVD comparing treatment and placebo group, and *Y*_*i*2_ denote the log hazard ratio of risk of stroke. Because CVD and stroke share many of risk factors, the two outcomes *Y*_*i*1_ and *Y*_*i*2_ are positively correlated. The within‐study correlations of *Y*_*i*1_ and *Y*_*i*2_ are presented in the right column of Figure 5. We conduct meta‐analyses of this data using the BRMA (REML), BRMA (Jackson) and the MMoM and estimate the overall composite effect of the antihypertensive treatment, as *δ* = (*β*_1_+*β*_2_)/2. The lower panel of Figure 5 presents the results from the three methods. The point estimate of *δ* is estimated as −0.312 (95*%* CI: (−0.432,−0.192)) by the BRMA (REML), and −0.305 (95*%* CI: (−0.430,−0.180)) by BRMA (Jackson) method. The MMoM provides very similar point estimate of *δ* as −0.313 but a slightly narrower 95*%* confidence interval as (−0.418,−0.208). Despite this, the proposed MMoM has made appropriate inferences for *δ* = (*β*_1_+*β*_2_)/2 for this example where the pairs of estimates from each study are correlated.

We also conduct a bivariate meta‐regression to further evaluate the treatment effects in SBP and DBP. Let ISH denote the indicator of trials included only isolated systolic hypertension patients (i.e. subjects with high SBP but normal DBP). We include ISH as a covariate for both outcomes in this meta‐regression, in order to allow studies of this type to estimate different effects. We now let *Y*_*i*1_ denote the difference in SBP between the treatment and the control groups, and *Y*_*i*2_ denote the difference in DBP between the treatment and the control groups. Let *β*_10_ and *β*_20_ denote the intercept in the meta‐regression model for *Y*_*i*1_ and *Y*_*i*2_, and *β*_11_ and *β*_21_ denote the regression coefficients associated with ISH for *Y*_*i*1_ and *Y*_*i*2_, respectively. It is of clinical importance to evaluate the overall composite effect of the reduction in SBP and DPB in patients with high SBP or high DBP, that is, trials where ISH = 0, so that our target for inference is the parameter *δ* = (*β*_10_+*β*_20_)/2. The point estimate of *δ* is estimated as −7.34 (95*%* CI: (−7.99,−6.69)) by the MMoM.

### 5.2 Example 2: School‐based programmes for smoking prevention

This example is taken from the Cochrane review ‘School‐based programmes for preventing smoking’ 35. The intervention of the study is a smoking prevention program by offering curricula to school students. Analysis 1.1 of this review compares the effectiveness of all curricula versus control for pure prevention cohorts (Cohorts in which never‐smokers at baseline were followed up), where the outcome of interest is whether subjects begin smoking and the follow up time is less than one year. Analysis 1.2 is as Analysis 1.1 but for this second analysis the follow up time is the longest available follow‐up. Hence, these two analyses address very similar questions, but Analysis 1.2 is intended to describe the effectiveness of all curricula over the longer term. Seventy three studies contribute outcome data to these analyses, which were performed on the log odds ratio scale. The pooled results were, however, presented on the odds ratio scale, where an odds ratio of less than one favours the intervention.

All 73 studies contribute to Analysis 1.2 for which the Cochrane review reports a pooled odds ratio of 0.88 (with a 95% confidence interval of [0.82, 0.96]), suggesting that intervention is effective in the longer term. Forty of these studies also contribute data to Analysis 1.1 for which the Cochrane review reports an odds ratio 0.94 (with a 95% confidence interval of [0.85, 1.05]), so there is no evidence of an intervention effect in the shorter term. In Analysis 1.1, the estimated between‐study variance was zero, and so, the random‐effects model collapses to a fixed effect model, and Analysis 1.1 was presented as a fixed effect model in the Cochrane review.

The confidence intervals from these two univariate analyses considerably overlap, but the outcomes are highly correlated; in some studies the estimates for both analyses are the same because their longest follow‐up was less than 1 year. The results presented in the Cochrane review, therefore, do not make it clear whether there is any evidence of a different treatment effect in the longer term. However, our method can be used to answer this question by making inference about *δ* = *β*_1_−*β*_2_ as in the simulation study.

The results from the MMoM estimate the difference in treatment effects of intervention between shorter term and longer term as *δ* = 0.064 (with a 95% confidence interval of [−0.048, 0.18]). Thus, there is no evidence of different treatment effect of intervention in shorter term and longer term. Because the BRMA (REML) and BRMA (Jackson) cannot be applied when the within‐study correlations are unknown, we impute three different nonnegative values of the within‐study correlations for all studies and compare the estimation of *δ* in Table 1. All three estimation methods show no evidence of a different treatment effect in the longer term. The point estimation from Jackson's method when the imputed within‐study correlations are large (i.e. 0.8) is close to that of MMoM method, while the confidence intervals from Jackson's method are narrower. We observe that the estimation from the REML and Jackson methods produce notably different findings for the three different imputed within‐study correlations *ρ*_*w*_, indicating that the performances of the REML and Jackson methods are sensitive to the value of imputed within‐study correlations. This example nicely illustrates how our proposed MMOM can be used to make further inferences from systematic reviews that use multiple univariate meta‐analyses where studies contribute data to more than a single meta‐analysis.

**Table 1:** The difference in treatment effect (*δ*) estimated by REML, Jackson and MMoM with imputed within‐study correlation for the Cochrane review ‘School‐based programmes for smoking prevention’.

*ρ*_*w*_	REML	Jackson	MMoM
0	0.08(−0.05, 0.22)	0.09(−0.05, 0.22)	0.06(−0.05, 0.18)
0.5	0.07(−0.02, 0.18)	0.07(−0.02, 0.17)	
0.8	0.07(−0.01, 0.16)	0.06(−0.01, 0.14)	

## 6 Discussion

We have proposed an extension of the standard procedure for univariate meta‐analysis. Our procedure uses conventional methods for univariate meta‐analysis for making inferences about the marginal effects but augments these univariate analyses with a covariance matrix for the resulting pooled estimates. Our approach has a variety of advantages. Firstly, the often unknown within‐study variances are not required when using our method. Secondly, our approach builds upon the univariate procedures that meta‐analysis will already be familiar with. Thirdly, very few estimation problems are encountered when using method. Finally, and perhaps most importantly, our procedure enables consumers of systematic reviews that perform multiple univariate meta‐analyses to make valid joint inferences and also valid inferences for any functions of the pooled estimates that might be of interest. The main disadvantage of our proposal is that no borrowing of strength is possible when using our new approach.

Another issue is that the proposed method is a non‐likelihood‐based frequentist method and so requires the MCAR assumption when any outcome data are missing. Some form of inverse probability weighting might be developed in order to make the weaker missing at random (MAR) assumption. Dealing with outcome reporting bias and publication bias is in general much more complicated, however, because these lead to missing not at random (MNAR) models. Because the proposed method is not based on the likelihood, complex modelling of this type in conjunction with our estimation procedure is, at best, not straightforward. The computation for handling missing data is very straightforward, but the necessity of the MCAR assumption must not be forgotten.

There are several potential topics of future. The extension to multivariate meta‐regression models is straightforward, and some models for network meta‐analysis can be fitted as regression models 36. Hence, the proposed approach may also be useful in the network meta analysis setting in datasets where the within‐study correlations are not available. In this paper, we apply the proposed method to multivariate meta‐analysis with two outcome. When applying the REML method to the multivariate setting with more than two outcomes, because to estimate. In contrast, the extension of the proposed method to meta‐analysis with more than two outcomes is straightforward and is less prone to computational issues. The empirical performance of the proposed method under multivariate setting with more than two outcomes will be investigated in the future.

The methodology builds upon standard univariate methods, and more sophisticated methods for univariate meta‐analysis could also be used conjunction with our approach. For example, confidence intervals for the between‐study variance in random effects meta‐analysis 37, and meta‐regression models 38 are immediately applicable. There is a vast univariate meta‐analysis literature, and by using standard univariate methods for the marginal inferences. the usefulness of all this literature is retained. Also our methodology is attractive from a more applied perspective, because all the results from the very many univariate meta‐analyses that have been performed to date and have informed medical practice are unchanged by our method. Hence, our method does not change anything that has been done already, rather it allows us to do more with published meta‐analytic results than was hitherto possible.

To summarize, we have developed a very simple and useful method for handling correlated outcome data in meta‐analysis when interest lies beyond making marginal inferences for each of the effects of interest. Our method has been found to perform well when compared with more sophisticated approaches and provides another useful tool for all those involved in performing and interpreting meta‐analyses.

## Supporting information



Supporting info item
