# Supplementary material for: Inference for correlated effect sizes using multiple univariate meta‐analyses
Source: Stat Med. 2015 Nov 4;35(9):1405–22. doi: 10.1002/sim.6789 (PMC4821787; doi:10.1002/sim.6789)
Supplement: Supplementary file 1 — Supporting info item [file SIM-35-1405-s001.pdf]

Research Article

Statistics in Medicine

Received XXXX

(www.interscience.wiley.com) DOI: 10.1002/sim.0000

Supplementary materials for "Inference for correlated effect sizes using multiple univariate meta-analyses"

In this supplementary material, we describe the use of the proposed method under missing data settings (Section 1) and prove the equivalence between two missing data approaches (Section 2), where the first one is to use the formulas derived in Section 1 of this supplemental material, and the second approach is to simply replace the missing outcomes by zero point estimates and large within-study variances. Furthermore, we provide the additional simulation results when the ratio of between-study variance and within-study variance equal to 0.4 and 1 (i.e. the between-study variations are $\tau_1^2 = \tau_2^2 = 0.1$ and 0.25 respectively) in Section 3. In Section 4, we provide an additional example to illustrate the application of the MMoM method in a meta-analysis where within-study correlations are unknown.

1. The proposed MMoM under missing data setting where only a subset of outcome are reported

To simplify the presentation, we assume that two endpoints are of interest. Consider a meta-analysis of m studies where the first m_1 studies reported both endpoints, the next m_2 studies reported the first endpoint only, and the remaining m_3 studies reported the second endpoint only. We assume missing completely at random. The Q statistic can be written as

$$\begin{aligned} Q_1 &= \sum_{i=1}^{m_1+m_2} \sigma_{i1}^{-2}(Y_{i1} - \bar{Y}_1) \\ Q_2 &= \sum_{i=1}^{m_1} \sigma_{i2}^{-2}(Y_{i2} - \bar{Y}_2) + \sum_{i=m_1+m_2+1}^{m_1+m_2+m_3} \sigma_{i2}^{-2}(Y_{i2} - \bar{Y}_2), \end{aligned}$$

where $\bar{Y}_1 = \frac{\sum_{i=1}^{m_1+m_2} \sigma_{i1}^{-2} Y_{i1}}{\sum_{i=1}^{m_1+m_2} \sigma_{i1}^{-2}}$, and $\bar{Y}_2 = \frac{\sum_{i=1}^{m_1} \sigma_{i2}^{-2} Y_{i2} + \sum_{i=m_1+m_2+1}^{m_1+m_2+m_3} \sigma_{i2}^{-2} Y_{i2}}{\sum_{i=1}^{m_1} \sigma_{i2}^{-2} + \sum_{i=m_1+m_2+1}^{m_1+m_2+m_3} \sigma_{i2}^{-2}}$.

The expectation of Q is given by

$$\begin{aligned} E[Q_1] &= (m_1 + m_2 - 1) + \left(s_{11} - \frac{s_{12}}{s_{11}}\right)\tau_1^2, \\ E[Q_2] &= (m_1 + m_3 - 1) + \left(s_{21} - \frac{s_{22}}{s_{21}}\right)\tau_2^2, \end{aligned}$$

where $s_{1r} = \sum_{i=1}^{m_1+m_2} \sigma_{i1}^{-2r}$, and $s_{2r} = \sum_{i=1}^{m_1} \sigma_{i2}^{-2r} + \sum_{i=m_1+m_2+1}^{m_1+m_2+m_3} \sigma_{i2}^{-2r}$, which provides the DerSimonian and Laird moment estimator

$$\begin{aligned} \hat{\tau}_1^2 &= \max\left\{0, \frac{Q_1 - (m_1 + m_2 - 1)}{s_{11} - \frac{s_{12}}{s_{11}}}\right\}, \\ \hat{\tau}_2^2 &= \max\left\{0, \frac{Q_2 - (m_1 + m_3 - 1)}{s_{21} - \frac{s_{22}}{s_{21}}}\right\}. \end{aligned}$$

Copyright © 2013 John Wiley & Sons, Ltd.
Prepared using simauth.cls [Version: 2010/03/10 v3.00]

The point estimate of the overall effect size can be obtained from univariate method used by DerSimonian and Larid, which are given as

$$\hat{\beta}_1 = \frac{\sum_{i=1}^{m_1+m_2} w_{i1} Y_{i1}}{\sum_{i=1}^{m_1+m_2} w_{i1}} \quad \text{and} \quad \hat{\beta}_2 = \frac{\sum_{i=1}^{m_1} w_{i2} Y_{i2} + \sum_{i=m_1+m_2+1}^{m_1+m_2+m_3} w_{i2} Y_{i2}}{\sum_{i=1}^{m_1} w_{i2} + \sum_{i=m_1+m_2+1}^{m_1+m_2+m_3} w_{i2}}$$

where

$$w_{i1} = (s_{i1}^2 + \hat{\tau_1}^2)^{-1} \quad \text{and} \quad w_{i2} = (s_{i2}^2 + \hat{\tau_2}^2)^{-1}.$$

$\hat{\beta}_1$ and $\hat{\beta}_2$ can be shown approximately normally distributed with covariance matrix

$$\Sigma_M = \begin{pmatrix} \left(\sum_{i=1}^{m_1+m_2} w_{i1}\right)^{-1} & \sum_{i=1}^{m_1} \frac{w_{i1}}{\sum_{i=1}^{m_1+m_2} w_{i1}} \frac{w_{i2}}{\sum_{i=1}^{m_1} w_{i2} + \sum_{i=m_1+m_2+1}^{m_1+m_2+m_3} w_{i2}} \mathrm{cov}(Y_{i1}, Y_{i2}) \\ & \left(\sum_{i=1}^{m_1} w_{i2} + \sum_{i=m_1+m_2+1}^{m_1+m_2+m_3} w_{i2}\right)^{-1} \end{pmatrix}.$$

2. Proof of the equivalence between two missing data approaches

By incorporating very large within-study variances to the missing observations, and setting the missing study outcomes to be 0, the Q statistic can be written as

$$Q_1^* = \sum_{i=1}^{m_1+m_2} \sigma_{i1}^{-2}(Y_{i1} - \bar{Y}_1) + \sum_{i=m_1+m_2+1}^{m_1+m_2+m_3} \sigma_{i1}^{-2}(Y_{i1} - \bar{Y}_1) \approx \sum_{i=1}^{m_1+m_2} \sigma_{i1}^{-2}(Y_{i1} - \bar{Y}_1)$$

where we use the $*$ notation to mean that missing observations have been incorporated in this way. Similarly,

$$Q_2^* \approx \sum_{i=1}^{m_1} \sigma_{i2}^{-2}(Y_{i2} - \bar{Y}_2) + \sum_{i=m_1+m_2+1}^{m_1+m_2+m_3} \sigma_{i2}^{-2}(Y_{i2} - \bar{Y}_2)$$

Therefore, $\hat{\tau}_j^{*2} \approx \hat{\tau}_j^2$.

$$\hat{\beta}_1^* = \frac{\sum_{i=1}^{m_1+m_2} w_{i1} Y_{i1} + \sum_{i=m_1+m_2+1}^{m_1+m_2+m_3} w_{i1} Y_{i1}}{\sum_{i=1}^{m_1+m_2} w_{i1} + \sum_{i=m_1+m_2+1}^{m_1+m_2+m3} w_{i1}} \approx \frac{\sum_{i=1}^{m_1+m_2} w_{i1} Y_{i1}}{\sum_{i=1}^{m_1+m_2} w_{i1}} = \hat{\beta}_1$$

Similarly,

$$\hat{\beta}_2^* \approx \hat{\beta}_2$$

Let $N = m_1 + m_2 + m_3$,

$$\Sigma_M^* = \begin{pmatrix} \left(\sum_{i=1}^{N} w_{i1}\right)^{-1} & \sum_{i=1}^{N} \frac{w_{i1}}{\sum_{i=1}^{N} w_{i1}} \frac{w_{i2}}{\sum_{i=1}^{N} w_{i2}} \mathrm{cov}(Y_{i1}, Y_{i2}) \\ & \left(\sum_{i=1}^{N} w_{i2}\right)^{-1} \end{pmatrix},$$

where

$$\sum_{i=1}^{N} w_{i1} = \sum_{i=1}^{m_1+m_2} w_{i1} + \sum_{i=m_1+m_2+1}^{m_1+m_2+m_3} w_{i1} \approx \sum_{i=1}^{m_1+m_2} w_{i1},$$

$$\sum_{i=1}^{N} w_{i2} \approx \sum_{i=1}^{m_1} w_{i2} + \sum_{i=m_1+m_2+1}^{m_1+m_2+m_3} w_{i2},$$

Copyright © 2013 John Wiley & Sons, Ltd.

$$
\begin{aligned}
&\sum_{i=1}^{N} \frac{w_{i1}}{\sum_{i=1}^{N} w_{i1}} \frac{w_{i2}}{\sum_{i=1}^{N} w_{i2}} \mathrm{cov}(Y_{i1}, Y_{i2}) \\
=\ &\sum_{i=1}^{m_1} \frac{w_{i1}}{\sum_{i=1}^{N} w_{i1}} \frac{w_{i2}}{\sum_{i=1}^{N} w_{i2}} \mathrm{cov}(Y_{i1}, Y_{i2}) + \sum_{i=m_1+1}^{m_1+m_2} \frac{w_{i1}}{\sum_{i=1}^{N} w_{i1}} \frac{w_{i2}}{\sum_{i=1}^{N} w_{i2}} \mathrm{cov}(Y_{i1}, Y_{i2}) \\
&+ \sum_{i=m_1+m_2+1}^{m_1+m_2+m_3} \frac{w_{i1}}{\sum_{i=1}^{N} w_{i1}} \frac{w_{i2}}{\sum_{i=1}^{N} w_{i2}} \mathrm{cov}(Y_{i1}, Y_{i2}) \\
\approx\ &\sum_{i=1}^{m_1} \frac{w_{i1}}{\sum_{i=1}^{N} w_{i1}} \frac{w_{i2}}{\sum_{i=1}^{N} w_{i2}} \mathrm{cov}(Y_{i1}, Y_{i2}) \\
\approx\ &\sum_{i=1}^{m_1} \frac{w_{i1}}{\sum_{i=1}^{m_1+m_2} w_{i1}} \frac{w_{i2}}{\sum_{i=1}^{m_1} w_{i2} + \sum_{i=m_1+m_2+1}^{m_1+m_2+m_3} w_{i2}} \mathrm{cov}(Y_{i1}, Y_{i2})
\end{aligned}
$$

Therefore, $\Sigma_M^* \approx \Sigma_M$.

3. Additional simulation results

The supplemental Figures 1-4 summarize the results when the ratio of between-study variance and within-study variance is close to 0.4, supplemental Figures 5-8 summarize the results when the ratio of between-study variance and within-study variance is close to 1 and supplemental Figures 9-12 summarize the results when the ratio of between and within-study variance is close to 2 and the within-study correlation are as extreme as -0.9 and 0.9. Each figure presents the empirical bias (EB), the coverage probability of nominal 95% confidence intervals (CP) and relative efficiency (RE) of the estimated difference between the effect sizes $\delta = \beta_1 - \beta_2$ using BRMA (REML), BRMA (Jackson) and the MMoM.

Figure 1 demonstrates the results when there is no missing data (referred to as the complete data setting) and the number of studies is small ($m = 10$). There is no evidence of bias in any of the simulation studies. The coverage probability of the proposed method is comparable to the alternative methods. The range of RE is $[96.9, 100.8]$ for Jackson's method and is $[95.7, 104.5]$ for the proposed method. This suggests that the MMoM is as good as the REML method and Jackson's method in terms of coverage and efficiency. Figure 2 presents the results when the number of studies is relatively large ($m = 25$). The coverage of the MMoM is around 90%, which is slightly better than Jackson's method but slightly poorer than the coverage of the BRMA (REML). The RE of the MMoM is close to 1 and is higher than the RE of Jackson's method. The results indicate that the BRMA (REML) performs slightly better in coverage and efficiency than MMoM when the study size is relatively large and the within-study correlations are available. Figure 3 summarizes the results in missing data setting (30% outcomes are missing), with number of studies is small ($n = 10$). The coverage probabilities of all three methods slightly decrease compared to the complete data setting. The coverage of Jackson's method is around 90% while that of BRMA (REML) and the MMoM are around 88%. The range of RE is $[96.9, 101.3]$ and $[96.0, 103.1]$ for Jackson's method and the MMoM respectively, which indicates that both MMoM and Jackson methods have similar efficiency as the BRMA (REML). Figure 4 presents the results when the number of studies is larger ($m = 25$) in missing data setting. The coverages of the three methods are close to each other and are improved compared to the coverages in Figure 3. The RE of the MMoM is slightly better than Jackson's method.

Figure 5 summarizes the results in the complete data setting when the ratio of between-study variance and within-study variance equal to 1, with number of studies is small ($n = 10$). The coverage probability of the MMoM is close to that of BRMA (REML) and is better than Jackson's method. The RE of the MMoM is comparable to that of Jackson's method. Figure 6 presents the results when the study size is larger ($n = 25$). The coverage probability of the MMoM method is around 90% and is robust to the between- and within-study correlations, whereas the coverage probability of Jackson's method deteriorates quickly as the between-study correlation becomes larger. The coverage of the BRMA (REML) is around 93% which is the best among three methods. The RE of the MMoM is close to 97% and is substantially better than that of Jackson's method. Figure 7 demonstrates the results when 30% of each outcome is missing and the study size is small ($n = 10$). The MMoM and Jackson's method produce similar coverage probabilities which are around 88% and are very similar to the coverage probabilities of BRMA (REML). The range of RE is $[97.5, 103.5]$ for the MMoM and is $[96.6, 102.6]$ for Jackson's method respectively, which indicates that the efficiency of the MMoM method is as good as Jackson's method.

Copyright © 2013 John Wiley & Sons, Ltd.

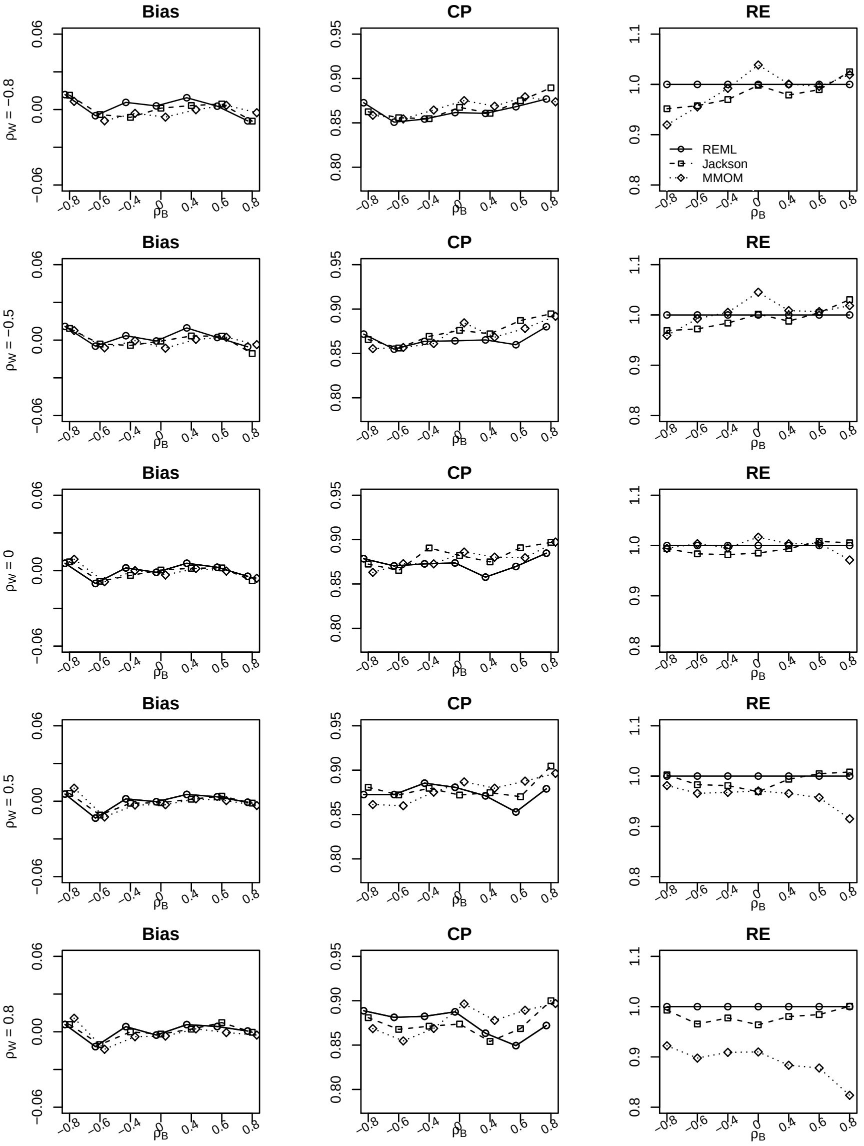

Figure 1. Bias, coverage probability (CP) and relative efficiency (RE) of $\delta = \beta_1 - \beta_2$, in 1,000 simulations based on data generated from BRMA model, with the between-study/within-study variation ratio close to 0.4, for number of studies m= 10, complete data setting and different between correlation ρ_B and within-study correlation ρ_{W_i}.

Figure 8 summarizes the results when number of studies is larger ($n = 25$) and data are missing. The coverage probabilities of all three methods are poorer than that in the complete data setting but the proposed MMoM appears to produce the best coverage probability among the three methods. The RE of the MMoM is around 98% which is substantially better than that of Jackson's method. By comparing the supplemental Figure 1-8 to the Figure 1-4 in main content, as the between-study variations become smaller (i.e., $\tau_1^2 = \tau_2^2 = 0.1$ or 0.25), all methods provide better coverages probabilities.

Figures 9 - 12 summarize the results when the within-study correlation are as extreme as -0.9 and 0.9. Figures 9-10 summarize the results in the complete data setting with study size equal to 10 and 25, respectively. Figures 11-12 summarize the results in the missing data setting with study size equal to 10 and 25, respectively. The coverage probabilities of all three methods are poorer than that when the within study correlation is in the normal range of (-0.8, 0.8) showing in Figures 1-4 in main content. The coverage probability of the MMoM is close to that of BRMA (REML) and is better than Jackson's method. The RE of the MMoM and the RE of the Jackson's method reduce dramatically when the within-study and between-study are both extreme ($\rho_b = 0.8, \rho_w = 0.9$). However, the RE of MMoM is substantially better than that of Jackson's method.

 Copyright © 2013 John Wiley & Sons, Ltd.

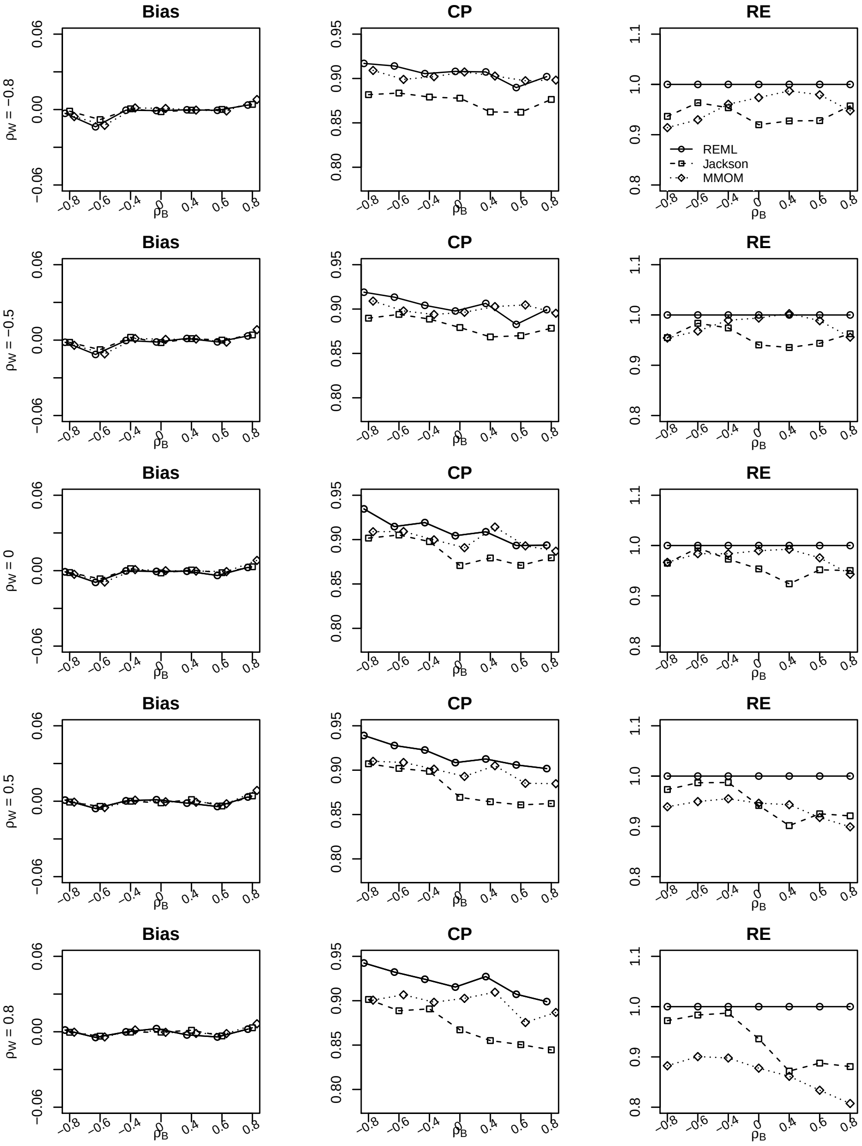

Figure 2. Bias, coverage probability (CP) and relative efficiency (RE) of $\delta = \beta_1 - \beta_2$, in 1,000 simulations based on data generated from BRMA model, with the between-study/within-study variation ratio close to 0.4, for number of studies $m = 25$, complete data setting and different between correlation ρ_B and within-study correlation ρ_{W_i}.

4. Data application : Mineral trioxide aggregate versus formocresol pulpotomy

Primary molars pulpotomy is operated when caries removal resulting in pulp exposure. Devitalization using formocresol and regenerating using mineral trioxide aggregate (MTA) are two widely used treatment approaches for pulpotomy. Recently, several safety issues related to utility of forocresol have been pointed out, and MTA has been recommended as a good alternative treatment for pulpotomy [1]. To compare the effectiveness of primary molars pulpotomy with MTA and formocresol, Shirvani and Asgary (2014) [1] performed a meta-analysis extracting the results from 16 clinical trials investigating the treatment effects of MTA and formocresol. Their results indicated that MTA was more effective than formocresol in primary molars pulpotomy with a lower failure rate for 6-, 12-, and 24-months follow-ups.

Besides reporting the relative risk of pulpotomy failure comparing MTA and forocresol for each time point of follow-ups, it is also of interest to evaluate the overall difference between log relative risks for different time points of follow-ups. For the ith study, let Y_{i1} denote the log relative risk of pulpotomy failure comparing MTA and forocresol for 12-months follow-up, Y_{i2} denote the same log relative risk as Y_{i1} but for 24-months follow-up. Here the log relative risks Y_{i1} and Y_{i2} are correlated but their correlations are not reported. Therefore the REML method

Copyright © 2013 John Wiley & Sons, Ltd.

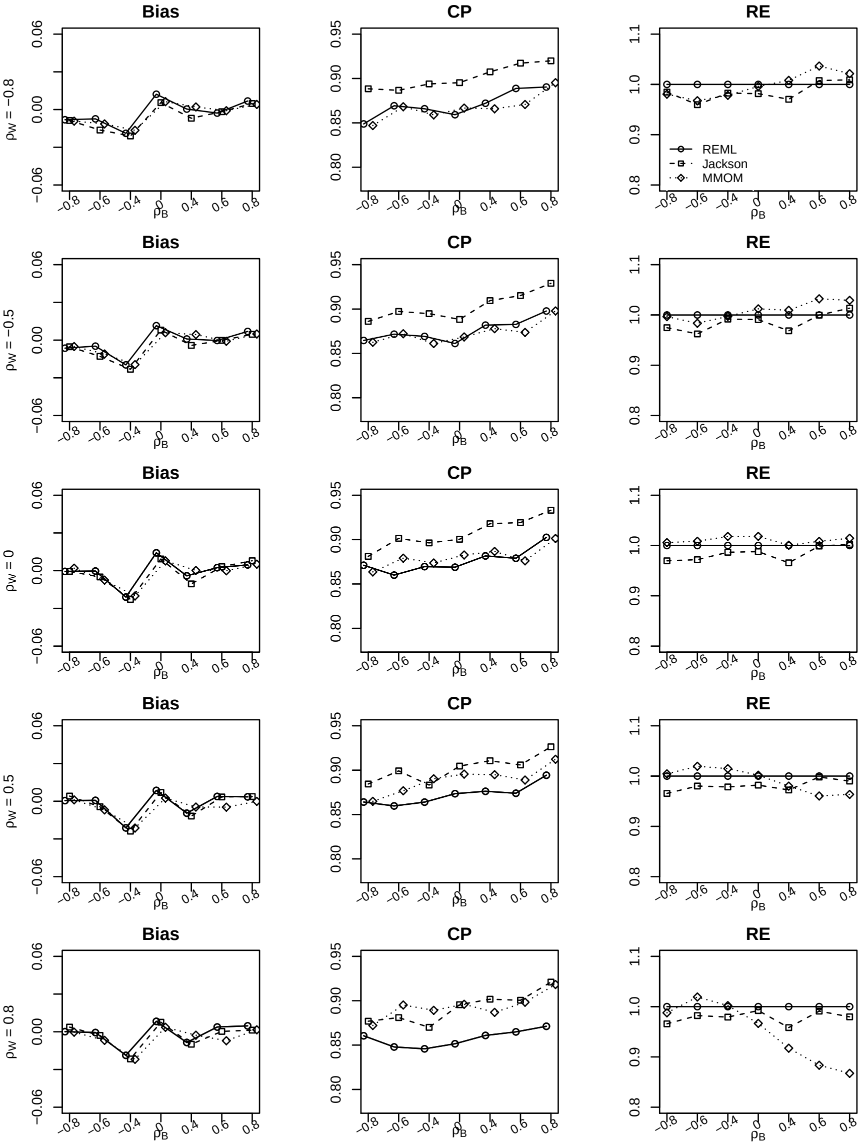

Figure 3. Bias, coverage probability (CP) and relative efficiency (RE) of $\delta = \beta_1 - \beta_2$, in $1,000$ simulations based on data generated from BRMA model, with the between-study/within-study variation ratio close to 0.4, for number of studies $m = 10$, missing data setting and different between correlation ρ_B and within-study correlation ρ_{W_i}.

and Jackson's method cannot be used. To evaluate the overall difference between the log relative risks for 12-months followup and those for 24-months followup, denoted as δ, we conduct a meta-analysis of 16 studies by using the MMoM. We note that only 6 studies have both outcomes reported, and the remaining 10 studies have only one of the two outcomes reported. Figure 13 presents the results from individual studies. Under the MCAR assumption, the MMoM can be applied to all 16 studies. The overall difference δ is estimated as -0.29 (95% CI: (-1.04, 0.45)) indicating that there is no significant difference between the log relative risk of pulpotomy failure comparing MTA and forocresol for 12-months follow-up and that for 24-months follow-up. We note that the MMoM leads to the same inferences for each outcome as univariate analysis. However, the MMoM joint inferences for all outcomes whereas the standard univariate method does not.

References

1. Shirvani A, Asgary S. Mineral trioxide aggregate versus formocresol pulpotomy: a systematic review and meta-analysis of randomized clinical trials. *Clinical oral investigations* 2014; **18**(4):1023–1030.

 Copyright © 2013 John Wiley & Sons, Ltd.

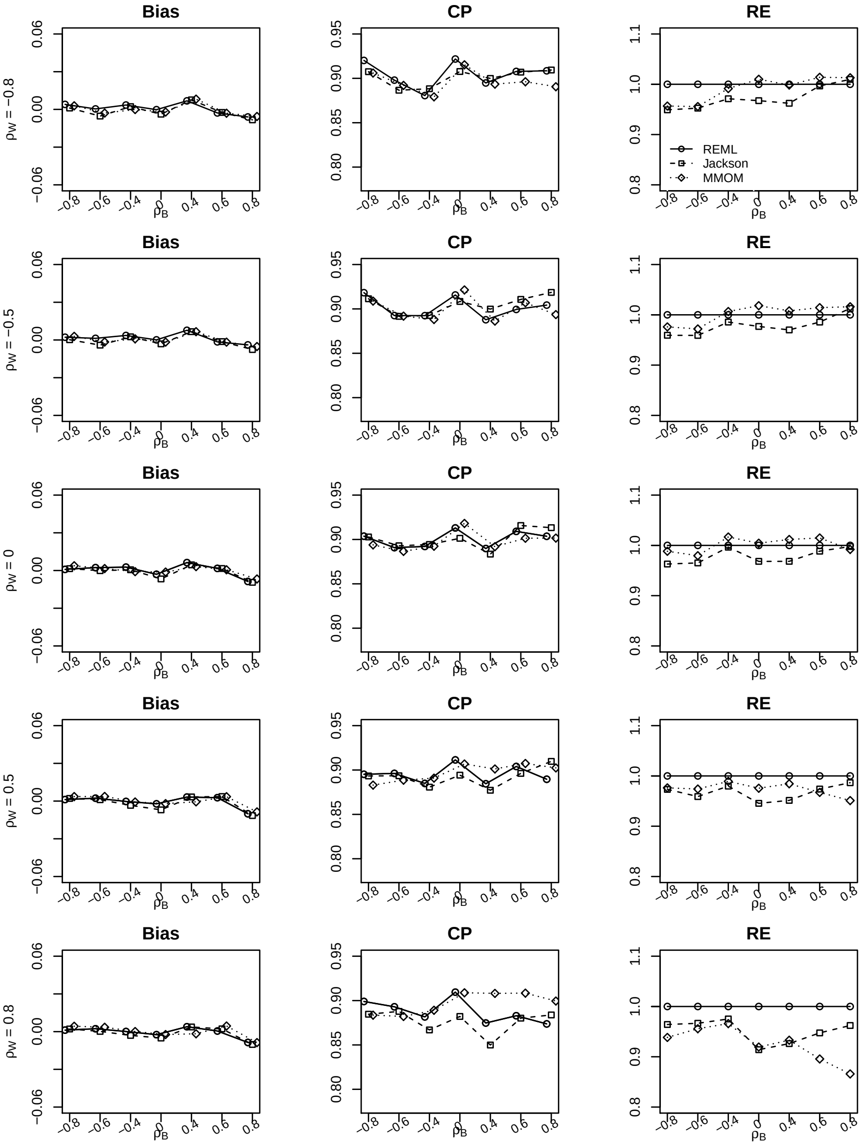

Figure 4. Bias, coverage probability (CP) and relative efficiency (RE) of $\delta = \beta_1 - \beta_2$, in $1,000$ simulations based on data generated from BRMA model, with the between-study/within-study variation ratio close to 0.4, for number of studies $m = 25$, missing data setting and different between correlation ρ_B and within-study correlation ρ_{W_i}.

Copyright © 2013 John Wiley & Sons, Ltd.

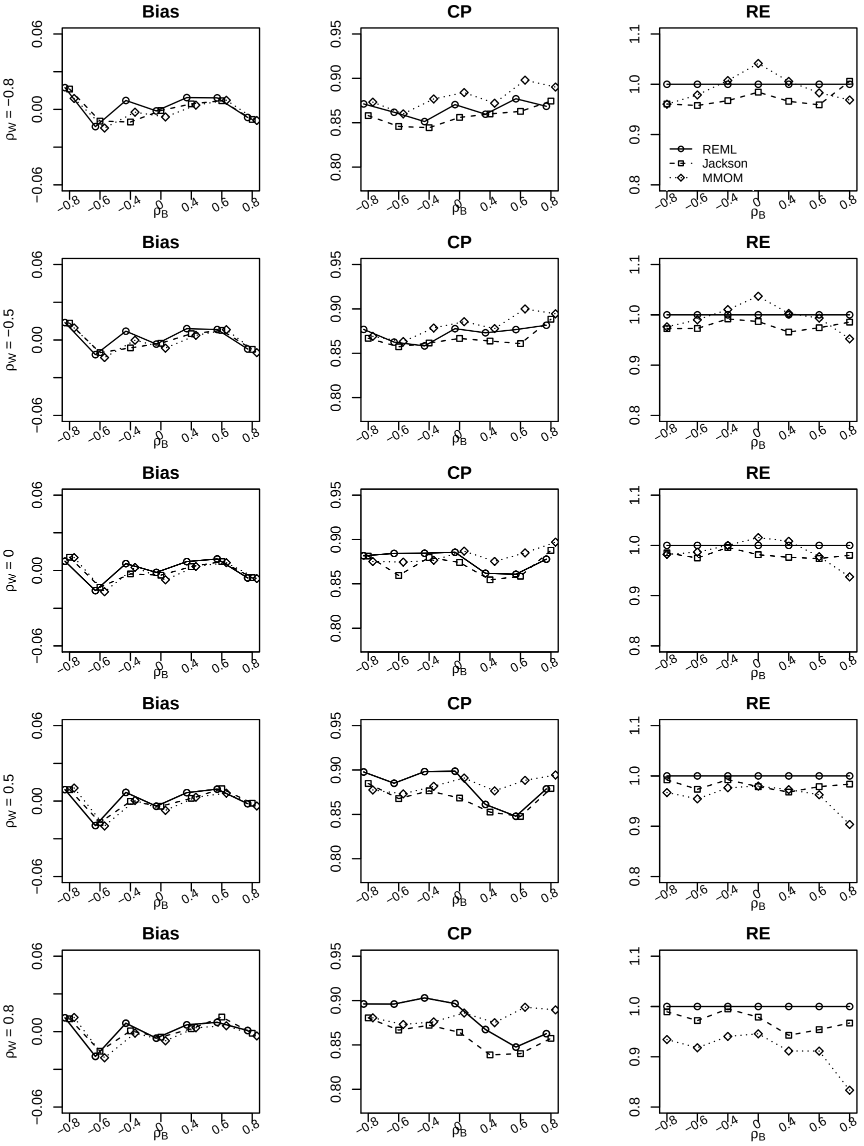

Figure 5. Bias, coverage probability (CP) and relative efficiency (RE) of $\delta = \beta_1 - \beta_2$, in $1,000$ simulations based on data generated from BRMA model, with the between-study/within-study variation ratio close to 1, for number of studies m= 10, complete data setting and different between correlation ρ_B and within-study correlation ρ_{W_i}.

 Copyright © 2013 John Wiley & Sons, Ltd.

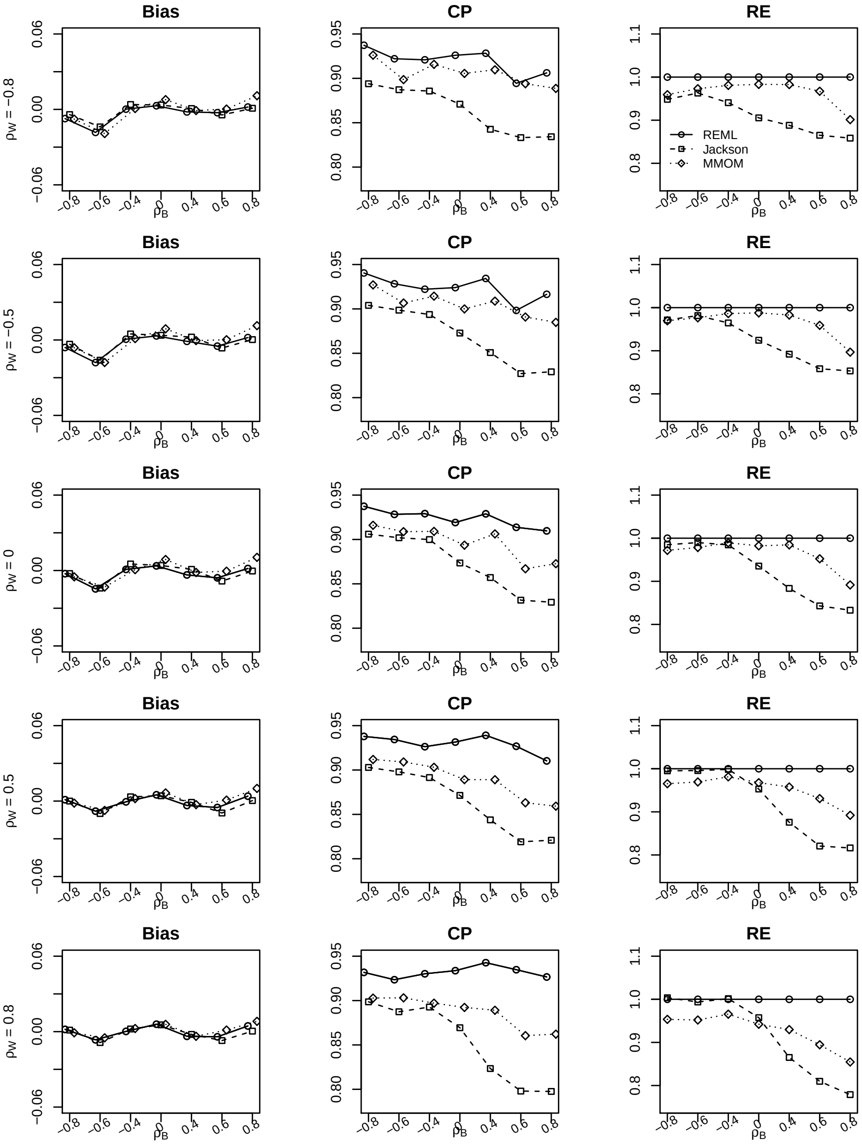

Figure 6. Bias, coverage probability (CP) and relative efficiency (RE) of $\delta = \beta_1 - \beta_2$, in 1,000 simulations based on data generated from BRMA model, with the between-study/within-study variation ratio close to 1, for number of studies $m = 25$, complete data setting and different between correlation ρ_B and within-study correlation ρ_{W_i}.

Copyright © 2013 John Wiley & Sons, Ltd.

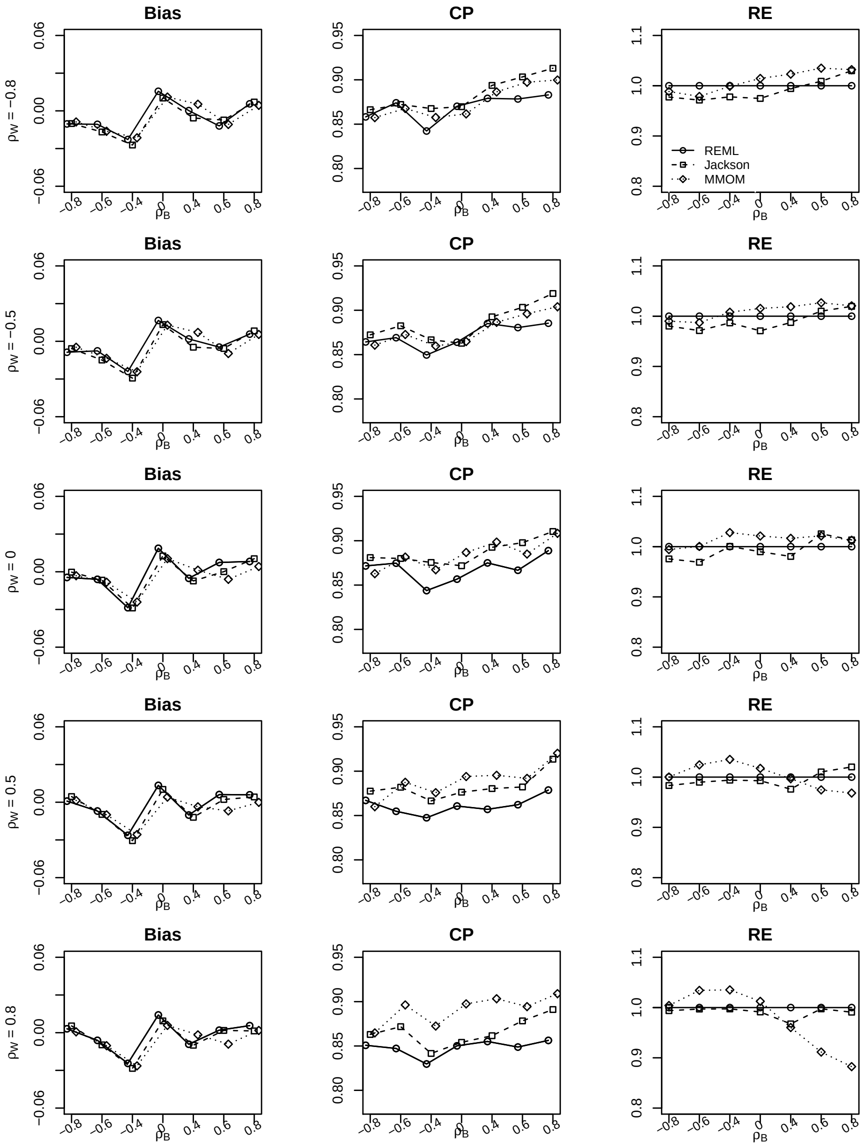

Figure 7. Bias, coverage probability (CP) and relative efficiency (RE) of $\delta = \beta_1 - \beta_2$, in $1,000$ simulations based on data generated from BRMA model, with the between-study/within-study variation ratio close to 1, for number of studies $m = 10$, missing data setting and different between correlation ρ_B and within-study correlation ρ_{W_i}.

 Copyright © 2013 John Wiley & Sons, Ltd.

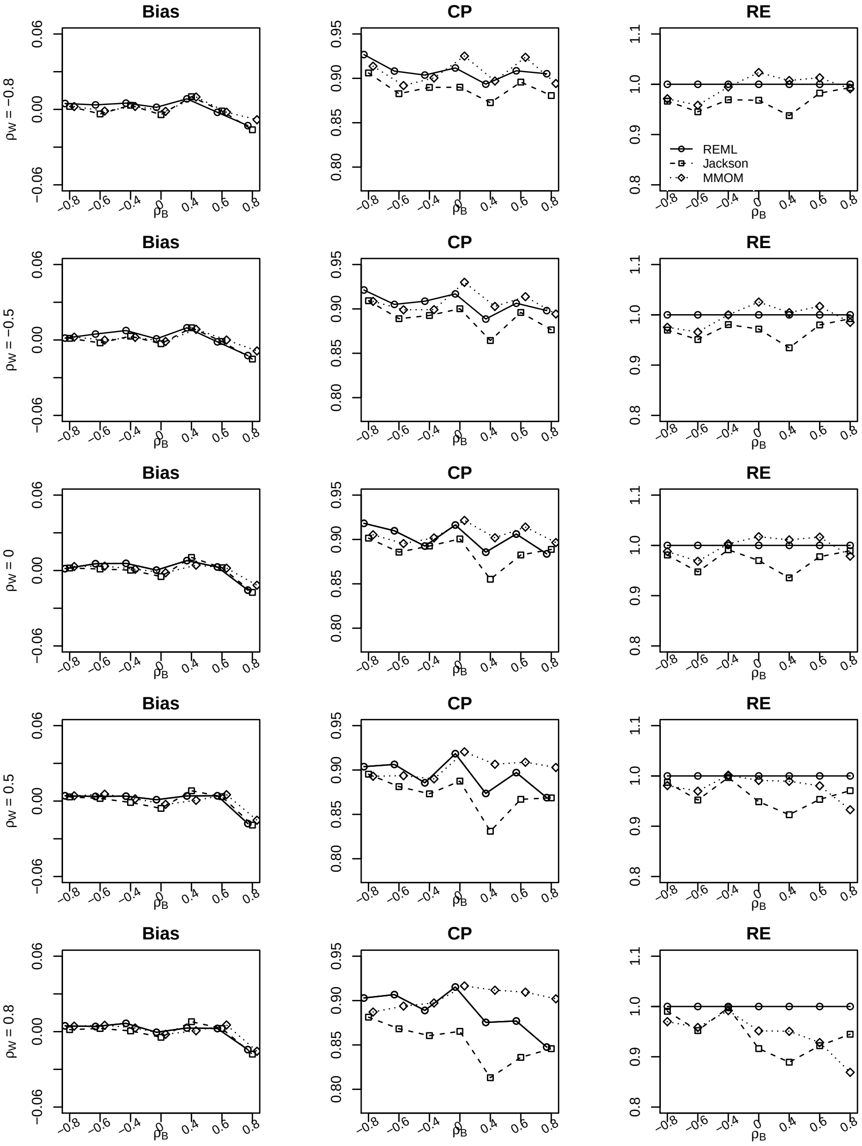

Figure 8. Bias, coverage probability (CP) and relative efficiency (RE) of $\delta = \beta_1 - \beta_2$, in 1,000 simulations based on data generated from BRMA model, with the between-study/within-study variation ratio close to 1, for number of studies $m = 25$, missing data setting and different between correlation ρ_B and within-study correlation ρ_{W_i}.

Copyright © 2013 John Wiley & Sons, Ltd.

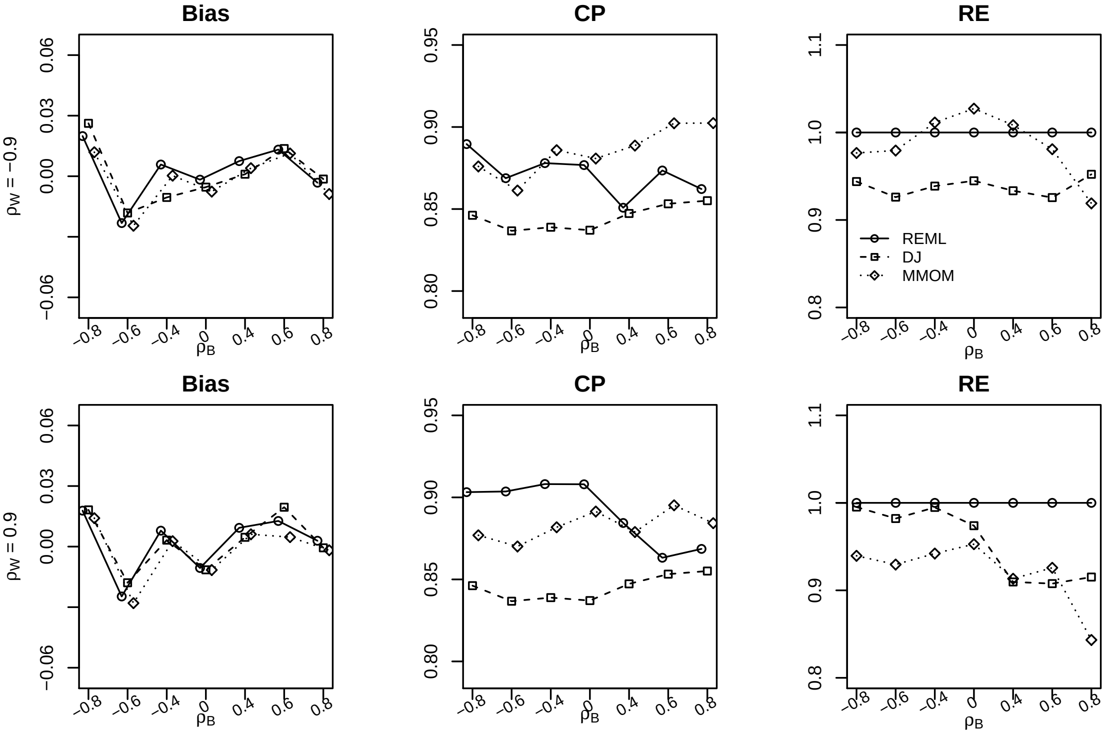

Figure 9. Bias, coverage probability (CP) and relative efficiency (RE) of $\delta = \beta_1 - \beta_2$, in 1,000 simulations based on data generated from BRMA model, with the between-study/within-study variation ratio close to 2, for number of studies $m = 10$, complete data setting and different between correlation ρ_B and with extreme within-study correlation $\rho_{W_i} = (-0.9, 0.9)$.

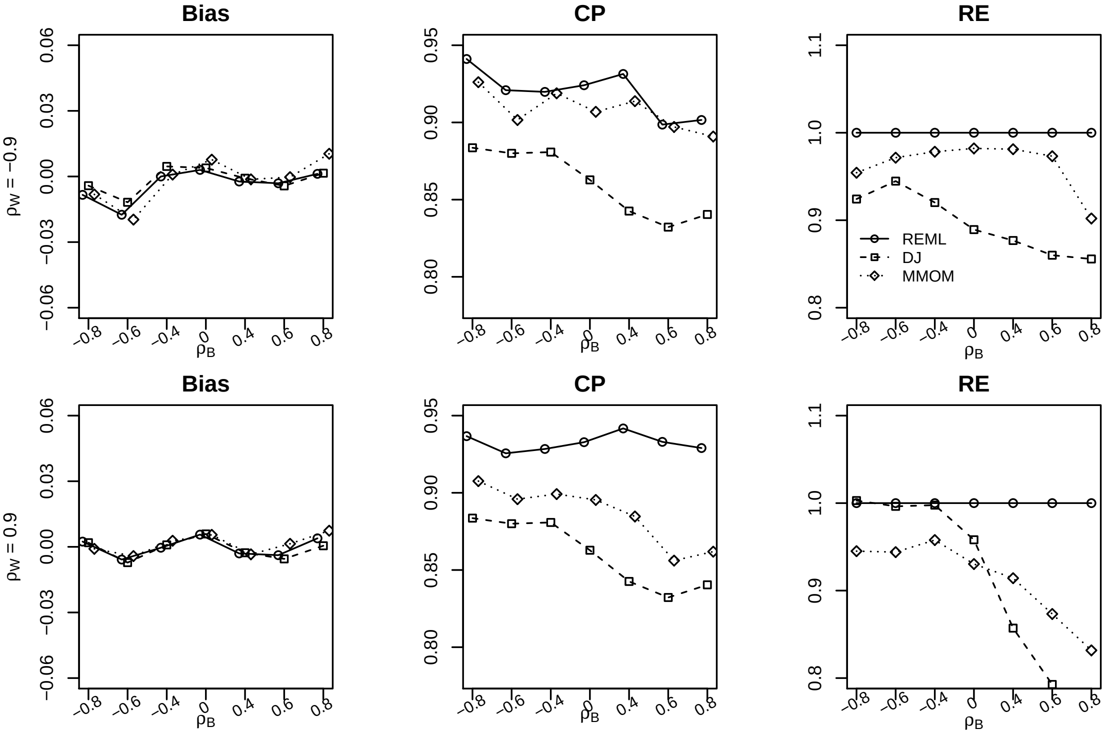

Figure 10. Bias, coverage probability (CP) and relative efficiency (RE) of $\delta = \beta_1 - \beta_2$, in 1,000 simulations based on data generated from BRMA model, with the between-study/within-study variation ratio close to 2, for number of studies $m = 25$, complete data setting and different between correlation ρ_B and with extreme within-study correlation $\rho_{W_i} = (-0.9, 0.9)$.

 Copyright © 2013 John Wiley & Sons, Ltd.

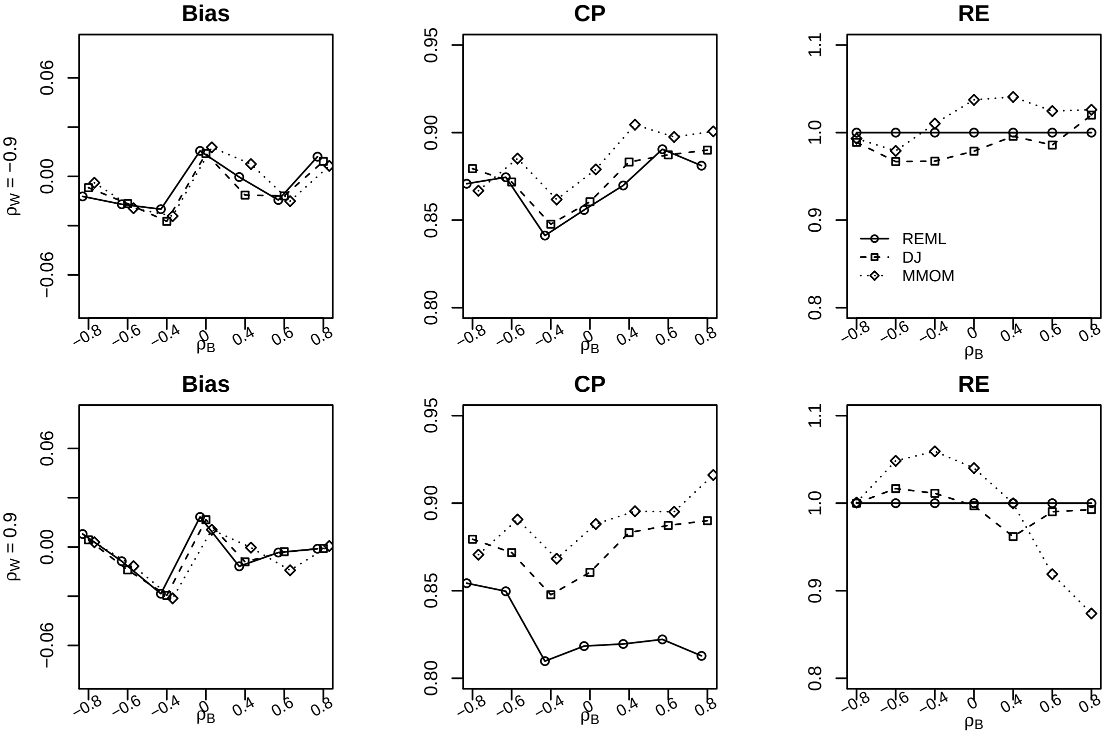

Figure 11. Bias, coverage probability (CP) and relative efficiency (RE) of $\delta = \beta_1 - \beta_2$, in 1,000 simulations based on data generated from BRMA model, with the between-study/within-study variation ratio close to 2, for number of studies $m = 10$, missing data setting and different between correlation ρ_B and with extreme within-study correlation $\rho_{W_i} = (-0.9, 0.9)$.

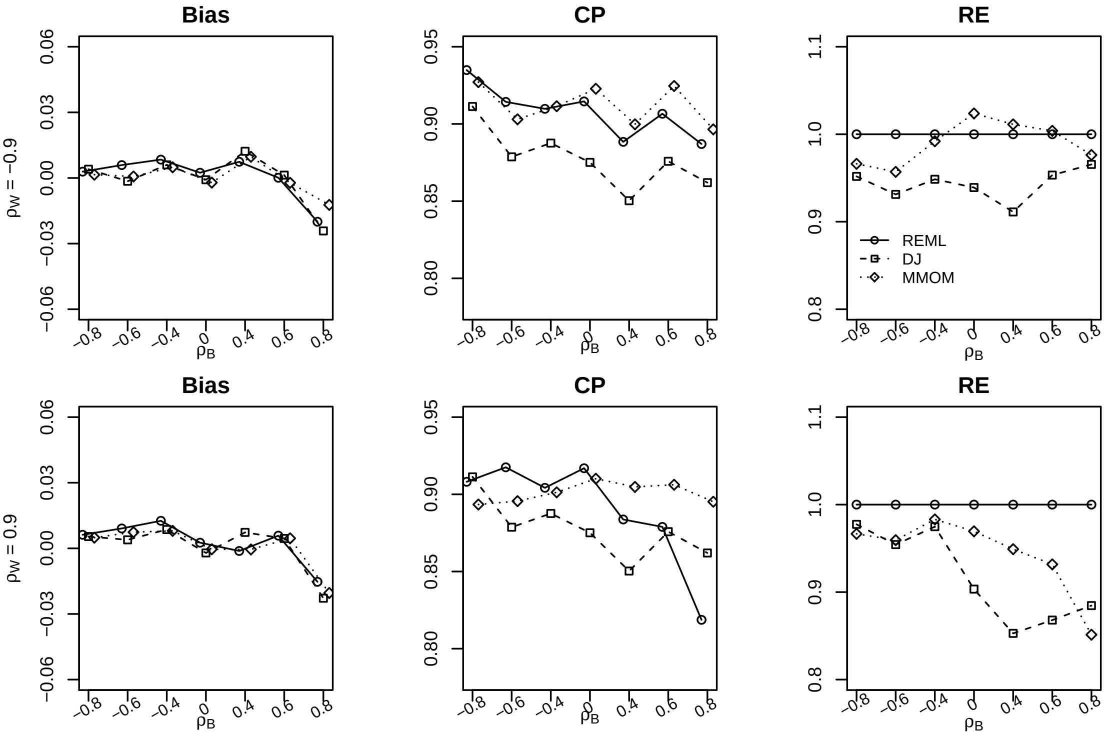

Figure 12. Bias, coverage probability (CP) and relative efficiency (RE) of $\delta = \beta_1 - \beta_2$, in 1,000 simulations based on data generated from BRMA model, with the between-study/within-study variation ratio close to 2, for number of studies $m = 25$, missing data setting and different between correlation ρ_B and with extreme within-study correlation $\rho_{W_i} = (-0.9, 0.9)$.

Copyright © 2013 John Wiley & Sons, Ltd.

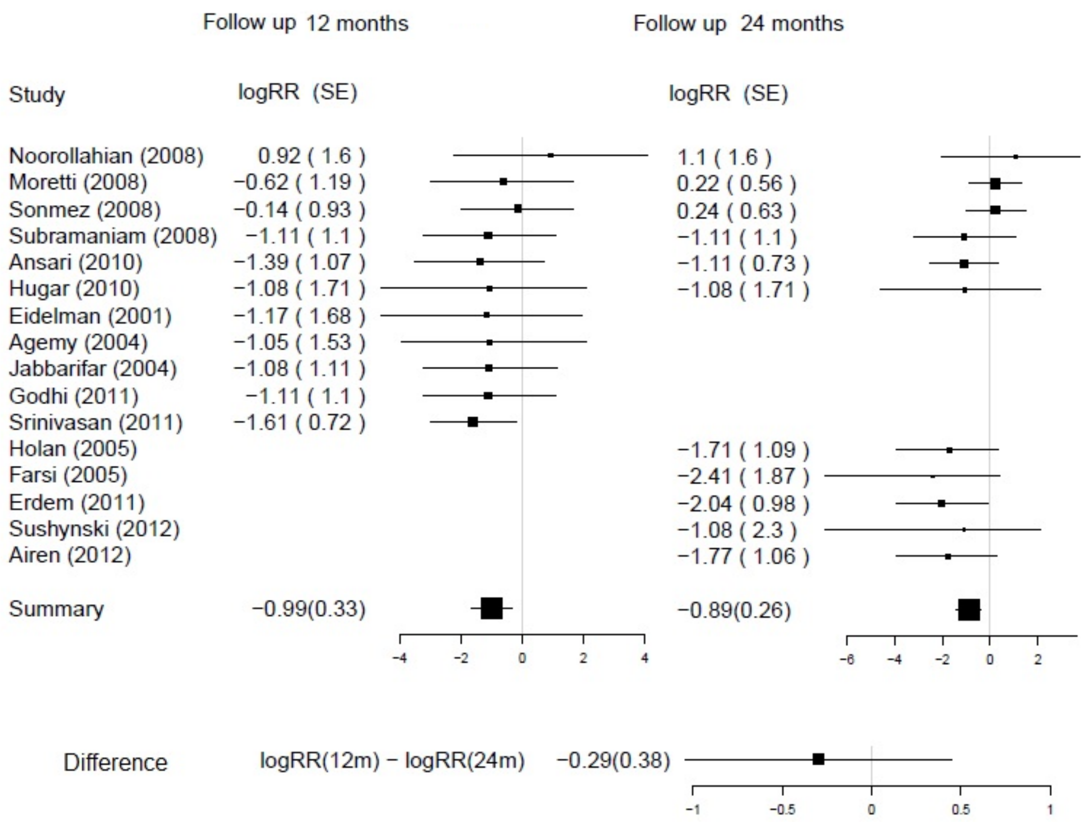

Figure 13. Differences between log relative risk for 12-months followup and for 24-months followup (δ) and 95% confidence intervals evaluated by the MMoM .

 Copyright © 2013 John Wiley & Sons, Ltd.
